# Catalytic asymmetric formal synthesis of beraprost

**DOI:** 10.3762/bjoc.11.285

**Published:** 2015-12-18

**Authors:** Yusuke Kobayashi, Ryuta Kuramoto, Yoshiji Takemoto

**Affiliations:** 1 Graduate School of Pharmaceutical Sciences, Kyoto University, Yoshida, Sakyo-ku, Kyoto 606-8501, Japan

**Keywords:** bifunctional catalysis, hydrogen bonding, organocatalyst, oxa-Michael, prostacyclin

## Abstract

The first catalytic asymmetric synthesis of the key intermediate for beraprost has been achieved through an enantioselective intramolecular oxa-Michael reaction of an α,β-unsaturated amide mediated by a newly developed benzothiadiazine catalyst. The Weinreb amide moiety and bromo substituent of the Michael adduct were utilized for the C–C bond formations to construct the scaffold. All four contiguous stereocenters of the tricyclic core were controlled via Rh-catalyzed stereoselective C–H insertion and the subsequent reduction from the convex face.

## Introduction

**Figure 1:**
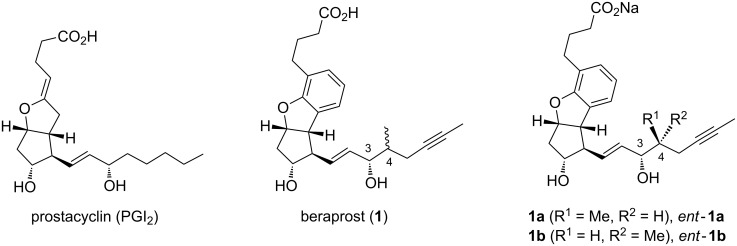
Structure of PGI_2_ and beraprost (**1**).

Prostacyclin (PGI_2_, Figure 1) is a physiologically active compound known to inhibit platelet activation and also acting as an effective vasodilator [1–3]. In addition to these properties, PGI_2_ derived from new vessels has attracted much attention due to its ability to promote axonal remodeling of injured neuronal networks after central nervous system disease [4–5]. However, PGI_2_ possesses an unstable enol ether moiety, which can be hydrolyzed even under neutral aqueous conditions, resulting in a loss of pharmacological action [6–8]. Therefore, an increasing number of more stable PGI_2_ derivatives have been developed. Among these, beraprost (**1**) has already been used as a pharmaceutical or under clinical trial in several countries for the treatment of arteriosclerosis obliterans and pulmonary hypertension [9]. Beraprost can be dosed orally as its sodium salt, and sold as a mixture of four diastereomers (**1a**, *ent*-**1a**, **1b**, and *ent*-**1b**) [10–13], although it was reported that each of the isomers have different activities [11]. In order to reduce the adverse effects while maintaining the pharmacological activities, an effective route for the asymmetric synthesis of **1** is highly sought after, and such methodologies should also lead to the expanded clinical application of **1**, as well as the development of more active derivatives.

**Scheme 1:**
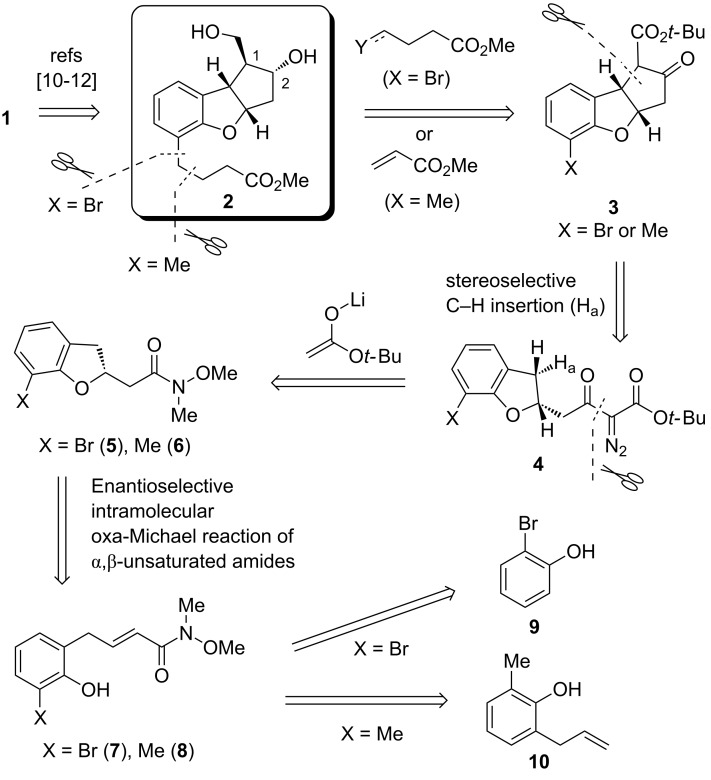
Retrosynthetic analysis of beraprost (**1**).

Due to the unique tricyclic core of **1**, which bears four contiguous stereocenters, various approaches for the synthesis of key intermediate **2** (Scheme 1) have been reported [14–23], including a few asymmetric syntheses relying on the optical resolution of racemic intermediates [16–18,23]. Herein we report the first catalytic asymmetric synthesis of the key intermediate **2** through organocatalyzed-enantioselective intramolecular oxa-Michael reaction [24–26].

## Results and Discussion

Our retrosynthetic analysis for **2** is shown in Scheme 1, with the derivatization of **2** to beraprost (**1**) having already been reported. We planned to introduce the ester side chain on the aromatic ring at a later stage, utilizing radical-mediated reactions with acrylate [22] when the functional group (X) at the *ortho* position was methyl, or via coupling reactions with C4 units when X was a bromo substituent. The *cis*-fused tricyclic core of **3** was assumed to be constructed by a stereoselective C–H insertion of diazoester **4**, which can be readily prepared from the Weinreb amides **5** or **6** via Claisen condensation followed by diazo-transfer reaction. The chiral dihydrobenzofuran scaffold (**5** or **6**) could be synthesized by asymmetric intramolecular oxa-Michael reaction (AIOM) of α,β-unsaturated amides **7** or **8**. Such reactions are generally considered to be challenging due to low nucleophilicity of the oxygen nucleophile and relatively unreactive Michael acceptors [27–33]. We envisioned that our recently developed powerful hydrogen bond (HB)-donor bifunctional organocatalyst [33] could promote the desired reaction of **7** or **8**, which can be synthesized from commercial sources **9** or **10**. Overall, the proposed strategy offers an efficient construction of all stereocenters of tricyclic core **2**, based on the initially established chiral stereocenter, as the configuration at the C1 and C2 positions of **2** would presumably be controlled by face-selective reduction of ketone **3**.

**Scheme 2:**
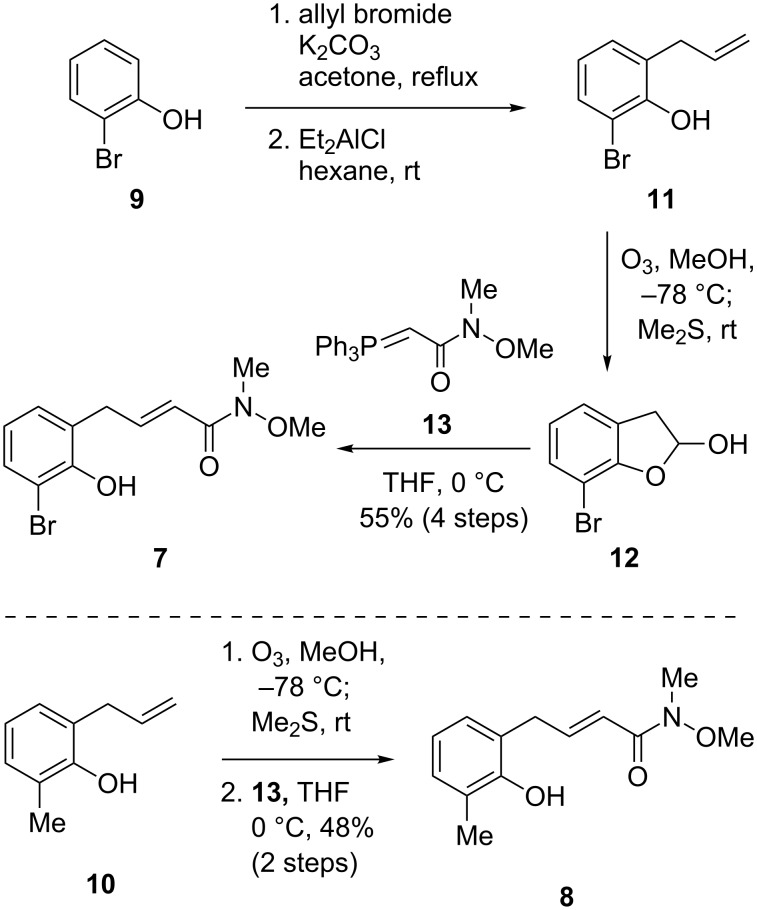
Preparation of Michael precursors **7** and **8**.

The Michael precursor **7** could be readily prepared from *ortho-*bromophenol (**9**, Scheme 2). *O*-Allylation of **9** followed by Lewis acid-mediated Claisen rearrangement afforded *ortho*-allylphenol **11**, whose olefin moiety was ozonolyzed and subsequently treated with Wittig reagent **13** to provide amide **7** in 55% yield over four steps from **9**. Amide **8** was similarly synthesized in 48% yield from **10**.

With the Michael precursors in hand, we next investigated the key AIOM reaction of **7** and **8** (Table 1). When the methylated substrate **7** was employed, the thiourea **B** [34–35] or benzothiadiazine **C** [33,36–38] catalysts efficiently promoted the reaction to furnish the dihydrobenzofuran **6** in 90% yield with high enantioselectivities (Table 1, entries 2 and 3). Conversely, thiourea **A** showed less catalytic activity, and the reaction required a much longer time for completion (Table 1, entry 1), indicating that the HB-donor moiety played an important role in facilitating the AIOM reaction. In addition, the AIOM reaction of bromo-substituted substrate **7** resulted in lower chemical yields (72–82%) and enantioselectivities (70–75% ee) even when catalysts **B** or **C** were employed for 72–120 hours (Table 1, entries 4 and 5). These results suggest that the relatively bulky bromo-substituent prevents recognition of the substrate by the catalyst. In order to improve recognition of the substrate through increased HB-donating abilities, we then tried catalyst **D** bearing a fluorine atom on the C6 position of the benzothiadiazine ring (Table 1, entry 6) [33]. As expected, both the chemical yield and enantioselectivity were improved, and the adduct **5** was obtained in 92% yield with 80% ee. In our preliminary DFT calculation, the HB-donor moiety would recognize an oxyanion generated from phenolic OH of substrates with tertiary amine moiety of the catalyst. It was also suggested that the SO_2_ moiety of benzothiadiazine catalyst would interact with the *N*-methyl substituent of the substrate by a non-classical hydrogen-bonding [39], improving the catalytic activities (see Supporting Information File 1 for details). The absolute configuration of **5** and **6** were assigned as (2*R*) by reference from the previous work [32–33]. Encouraged by this result, we next designed the new catalyst **E** with a stronger electron-withdrawing CF_3_ group on the aromatic ring, and applied it to the present AIOM reaction of **7** (Table 1, entry 7). To our delight, the enantioselectivity was improved to 85% ee while maintaining the high reactivity. Employing catalyst **E** we further investigated the reaction conditions and found that a scale-up synthesis can be performed using only 1 mol % of **E** with no loss of enantioselectivity, although gentle heating was required to ensure a high chemical yield (Table 1, entry 8).

**Table 1:** Optimization of asymmetric intramolecular oxa-Michael reaction.

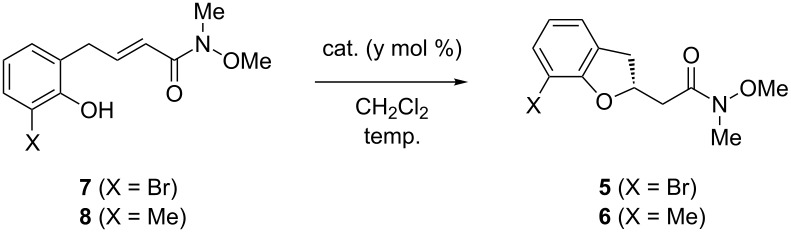							

entry	X	cat.	y	temp	time (h)	yield (%)^a^	ee (%)^b^

1	Me	**A**	10	rt	192	95	90
2	Me	**B**	10	rt	72	90	91
3	Me	**C**	10	rt	72	90	93
4	Br	**B**	10	rt	72	82	75
5	Br	**C**	10	rt	120	72	70
6	Br	**D**	10	rt	120	92	80
7	Br	**E**	10	rt	96	83	85
8	Br	**E**	1	35	96	93	86

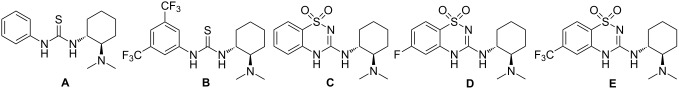
^a^Isolated yields. ^b^Determined by HPLC.

**Scheme 3:**
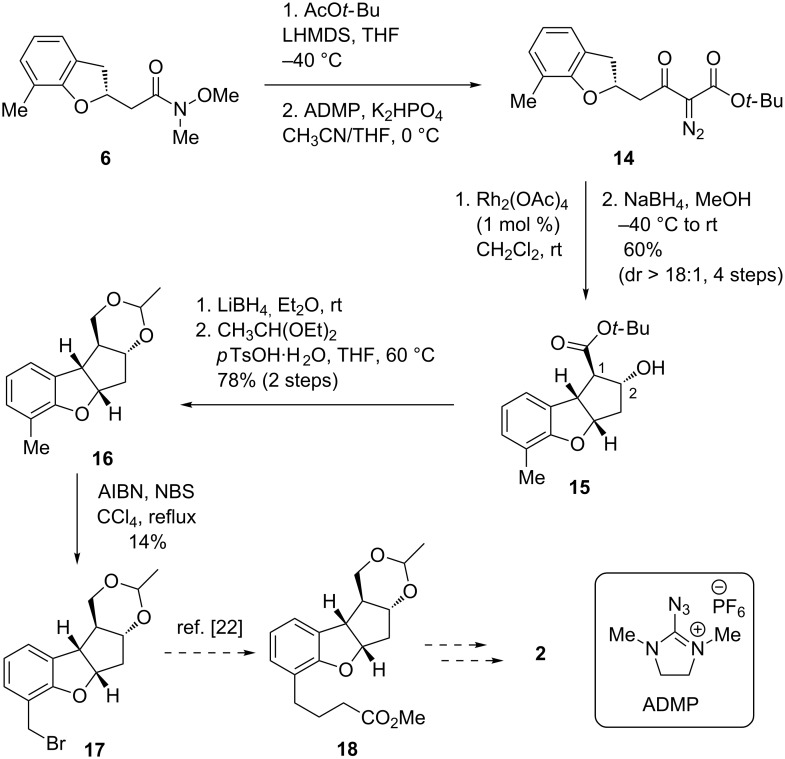
First attempt at the synthesis of **2** from **6**.

**Scheme 4:**
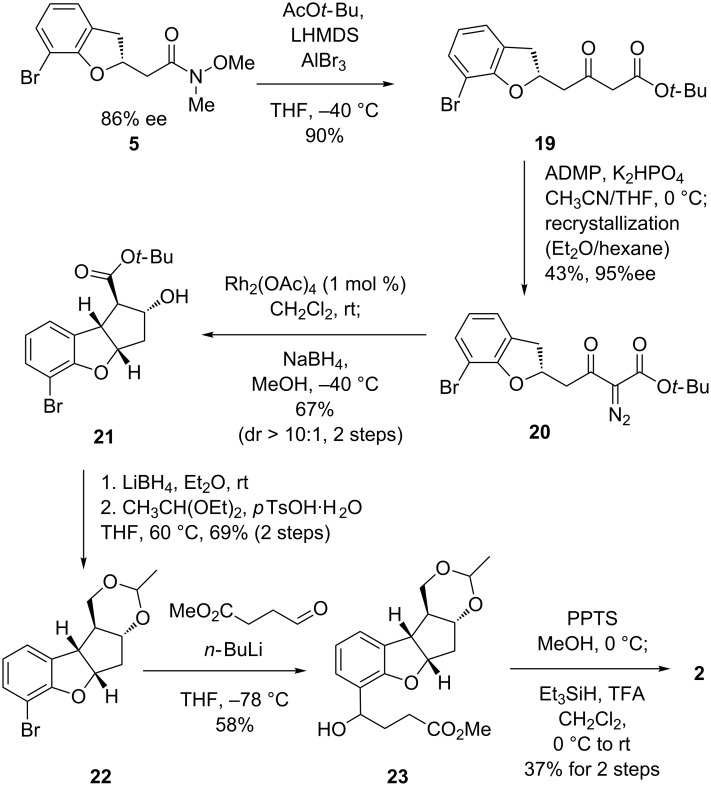
Achievement of a formal synthesis of **2**.

With both the AIOM adducts **5** and **6** in hand, we next investigated the construction of the tricyclic core (Scheme 3 and Scheme 4). The cross-Claisen condensation of **6** with lithium *tert*-butyl acetate afforded the corresponding β-ketoester, which was then treated with 2-azido-1,3-dimethylimidazolinium hexafluorophosphate (ADMP) [40–42] to give the diazoester **14**. Rhodium catalysed C–H insertion [43–44] of **14** proceeded smoothly to furnish the tricyclic ketoester, which was found to be unstable to purification on column chromatography, presumably due to decomposition of the ketoester moiety. Therefore, the product was isolated as alcohol **15** after a one-pot reduction of the ketone moiety (60% in four steps from **6**), along with the minor diastereomer at C1 position (dr > 18:1). These results mean that the stereochemistry at the C2 position was fully controlled, presumably due to hydride attack from the less-hindered convex face. The relative configuration of **15** was unambiguously determined by NOESY analysis (see Supporting Information File 1 for details). As all four desired stereocenters were constructed, we next investigated the introduction of the ester side chain on the aromatic ring via benzylic bromination followed by elongation of the C3 unit [22]. To this end, the ester group at the C1 position of **15** was reduced by lithium borohydride, and the resultant 1,3-diol protected to give acetal **16** [16]. After various experiments, selective bromination of the methyl group on the aromatic ring of **16**, however, was found to be difficult due to competitive bromination of the electron-rich aromatic ring, and thus the desired bromide **17** was obtained in only 14% yield.

We then investigated an alternative route from adduct **5**, even though the enantiomeric excess of **5** (86% ee) was a little lower than that of adduct **6** (93% ee) (Scheme 4). Fortunately the diazoester **20**, as similarly derived as in Scheme 3, was obtained as a crystalline solid, and one recrystallization increased the ee to 95%. The tricyclic scaffold **21** with all four stereocenters of the desired configuration was synthesized in 67% yield (dr > 10:1) via the method established in Scheme 3. After derivatization to acetal **22** in 2 steps, we then turned our attention to the introduction of the C4 ester substituents. Amongst various different conditions investigated – including Pd-catalyzed coupling reactions – a halogen-lithium exchange and subsequent addition to methyl 4-oxobutanoate was found to be the best method to introduce the C4 subunit with reproducibility in the case of scale-up synthesis. Deprotection of the resultant ester **23** followed by reduction of the benzylic OH group finally afforded the key intermediate **2**.

## Conclusion

We have developed the first asymmetric catalytic synthesis of the key intermediate for beraprost in 14 steps, via an organocatalyzed AIOM reaction of α,β-unsaturated amides. During the course of this study, it was revealed that a bromo substituent *ortho* to the phenolic OH group significantly decreased the reactivity and enantioselectivity. However, we found that the newly developed organocatalyst **E**, bearing increased HB-donating abilities, could improve both the reactivity and selectivity. In addition, the Weinreb amide moieties of the AIOM adduct were shown to be efficiently converted to β-ketoesters and diazoesters, a reactivity that could be further extended to various other molecular transformations. We believe that these findings could be applied to the synthesis of other biologically active oxo-heterocycles, and thus this is currently under investigation in our laboratory and will be reported in due course.

## Experimental

### General procedure for asymmetric oxa-Michael reaction

The benzothiadiazine catalyst **E** (8.6 mg, 0.022 mmol, 1 mol %) was added to a solution of **7** (661 mg, 2.20 mmol) in CH_2_Cl_2_ (20 mL), and the resulting mixture was stirred at 35 ºC for 96 h. The reaction mixture was then evaporated and the resulting crude residue purified by column chromatography on silica gel eluting with *n*-hexane/ethyl acetate (60/40) to give the analytically pure compound **5** (614 mg, 93%). The enantiomeric ratio was determined by HPLC on a chiral stationary phase (86% ee).

## Supporting Information

File 1 Experimental procedures and characterization data.
