# Supplementary material for: Catalytic asymmetric formal synthesis of beraprost
Source: Beilstein J Org Chem. 2015 Dec 18;11:2654–60. doi: 10.3762/bjoc.11.285 (PMC4685892; doi:10.3762/bjoc.11.285)
Supplement: File 1 — Experimental procedures and characterization data. [file Beilstein_J_Org_Chem-11-2654-s001.pdf]

Supporting Information

for

Catalytic asymmetric formal synthesis of beraprost

Yusuke Kobayashi, Ryuta Kuramoto, and Yoshiji Takemoto*

Address: Graduate School of Pharmaceutical Sciences, Kyoto University, Yoshida, Sakyo-ku, Kyoto 606-8501, Japan

Email: Yoshiji Takemoto - takemoto@pharm.kyoto-u.ac.jp

*Corresponding author

Experimental procedures and characterization data

CONTENTS

1. General information -- **S3**

2. Experimental details -- **S3**

3. Reference--- **S13**

4. Copies of ^{1}H and ^{13}C NMR charts--- **S14**

5. Copies of HPLC charts--- **S31**

6. Computational details -- **S35**

1. General information

All reactions were carried out under a positive atmosphere of argon in dried glassware. Analytical thin-layer chromatography was performed with Silica gel 60 (Merck). Column chromatography was performed on Merck silica gel 60 (230−400 mesh) or Fuji Silysia silica gel (NH, 100−200 mesh), and flash column chromatography was performed on Cica silica gel 60 (spherical/40−100 μm). Proton nuclear magnetic resonance (^{1}H NMR) spectra were recorded on a JEOL JNM-ECA 500 at 500 MHz, and AL400 at 400 MHz. Chemical shifts are reported relative to Me_4Si (δ 0.00) in $CDCl_3$. Multiplicity is indicated by one or more of the following: s (singlet); d (doublet); t (triplet); q (quartet); m (multiplet); br (broad). Carbon nuclear magnetic resonance (^{13}C NMR) spectra were recorded on a JEOL JNM-ECA 500 at 126 MHz, and AL400 at 100 MHz. Chemical shifts are reported relative to Me_4Si (δ 0.00) in $CDCl_3$ and internal residual solvent (MeOD-d_4 δ 3.31). Infrared spectra were recorded on a FT/IR-4100 Fourier-transform infrared spectrometer ATR (attenuated total reflectance). High-resolution mass spectra were obtained on a Shimazu LCMS-IT-TOF fitted with an ESI. Optical rotations were determined with a JASCO P-2200KDT polarimeter and are the average of five measurements. All melting points were measured on BÜCHI M-565 melting point apparatus and are uncorrected.

2. Experimental details

Preparation of catalyst

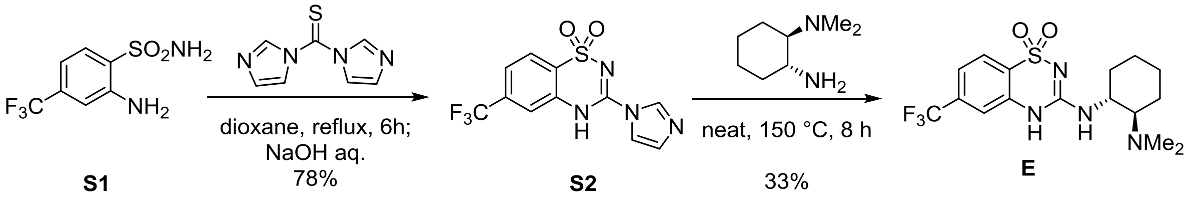

3-(1*H*-Imidazol-1-yl)-6-(trifluoromethyl)-4*H*-benzo[*e*][1,2,4]thiadiazine 1,1-dioxide (S2)

A solution of 2-amino-4-(trifluoromethyl)benzenesulfonamide [1] (**S1**, 2.97 g, 12.4 mmol) and *N*,*N*′-thiocarbonyldiimidazole (4.40 g, 24.7 mmol) in dioxane (50 mL) was refluxed for 5 h. The solvent was removed by distillation under reduced pressure, and the residue was suspended in water (50 mL). A solution of NaOH in water (2.0 g/20 mL) was added, and the solution was stirred at room temperature for 20 min. The alkaline solution was treated with 6 N HCl (40 mL). The precipitate was collected by filtration, washed with water, and dried to give the pale yellow solid of **S2** (3.07 g, 78%).

Mp: 180 °C (decomposed); ^{1}H NMR (500 MHz, $CDCl_3$): δ 9.74 (s, 1H), 8.22 (s, 1H), 7.93 (d, *J*=8.6 Hz, 1H), 7.75 (s, 1H), 7.57–7.58 (m, 2H) ppm, two N—H protons could not be observed; ^{13}C NMR (101 MHz, $CDCl_3$): δ 147.2, 145.2, 134.8, 131.9 (q, *J* = 31.8 Hz), 127.3, 124.3, 123.6 (q, *J* = 273 Hz), 122.1 (d, *J* = 4.8 Hz), 120.3 (q, *J* = 3.8 Hz), 119.3, 118.7 ppm; IR (Zn/Se-ATR) 3537, 3150, 1590, 1545, 1428, 1334 cm^{-1}; HRMS (ESI$^+$) Cacld. for $C_{11}H_8N_4O_2F_3S$ ($[M+H]^+$): 317.0315, found: 317.0329.

3-(((1*R*,2*R*)-2-(Dimethylamino)cyclohexyl)amino)-6-(trifluoromethyl)-4*H*-benzo[*e*][1,2,4]thiadiazine 1,1-dioxide (Catalyst E)

The mixture of **S2** (318.3 mg, 1.0 mmol) and (*R*, *R*)-N^1,N^1-dimethyl-*trans*-diaminocyclohexane [2] (213.4 mg, 1.5 mmol) was stirred at 150 °C for 6 h. The reaction mixture was dissolved in $CHCl_3$ (20 mL) and was washed with saturated aqueous NH_4Cl solution (10 mL) and saturated aqueous $NaHCO_3$ (10 mL). The organic layer dried over anhydrous Na_2SO_4. The solvent was evaporated and the resulting crude residue purified by column chromatography on silica gel (NH) eluting with chloroform/methanol (9/1) to afford **E** (128.6 mg, 33%) as brown amorphous.

^{1}H NMR (400 MHz, $CDCl_3$): δ 8.03 (d, *J* = 7.7 Hz, 1H), 7.47 (d, *J* = 7.4 Hz,1H), 7.07 (s, 1H), 3.45 (brs, 1H), 2.60–2.48 (m, 8H), 2.24 (brs, 1H), 2.00–1.98 (m, 1H), 1.89–1.87 (m, 1H), 1.80–1.77 (m, 1H), 1.32–1.23 (m, 5H), ppm; ^{13}C NMR (101 MHz, $CDCl_3$): δ 152.6, 137.8, 134.1 (q, *J* = 32.6 Hz), 125.2, 124.7, 123.0 (q, *J* = 273 Hz), 120.0 (d, *J* = 3.8 Hz), 114.6, 67.6, 52.8, 40.2, 32.8, 29.7, 24.4, 22.2 ppm; IR (Zn/Se-ATR) 3304, 2939, 1633, 1125 cm^{-1}; HRMS (ESI$^+$) Cacld. for $C_{16}H_{22}N_4O_2F_3S$ ([M+H]$^+$): 391.1410: found: 391.1391.

Catalytic asymmetric synthesis of important intermediate **2**

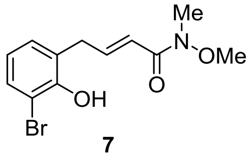

7

(*E*)-4-(3-Bromo-2-hydroxyphenyl)-*N*-methoxy-*N*-methylbut-2-enamide (7)

To the solution of *o*-bromophenol (**9**, 9.84 g, 56.9 mmol) and K_2CO_3 (15.3 g, 111 mmol) in acetone (125 mL) was added allyl bromide (10 g, 82.2 mmol), and the reaction mixture was refluxed at 70 °C for 2 h. The reaction mixture was filtered through a pad of Celite and the filtrate was evaporated under reduced pressure. The resulting mixture was extracted with EtOAc three times, and the combined organic layers were washed with brine, dried over Na_2SO_4, and concentrated under reduced pressure to give the crude allyl ether, which was used for the next reaction without further purification.

The crude ether (11.0 g) was dissolved in hexane (130 mL), and Et_2AlCl (1.0 M in hexane, 53 mL, 53 mmol) was added at room temperature. After being stirred at the same temperature for 3.5 h, the reaction mixture was cooled to 0 °C and quenched by careful addition of 2N HCl (35 mL). The aqueous phase was then separated and extracted with EtOAc three times. The combined organic layers were washed with brine, dried over Na_2SO_4, and concentrated under reduced pressure to afford 2-allyl-6-bromophenol (**11**, 12.7 g), which was directly subject to the next ozonolysis. A crude solution of **11** in MeOH (150 mL) was cooled to –78 °C, and O_3 gas was bubbled through the solution at –78 °C until the starting material had disappeared. The reaction was quenched with Me_2S (5.6 mL, 75.7 mmol), slowly warmed to room temperature before the addition of water (60 mL). The resulting mixture was stirred for 1 h and concentrated under reduced

pressure. The residue was diluted with EtOAc and washed with water. The organic layer was dried over Na_2SO_4 and concentrated in vacuo to give the crude aldehyde as a mixture with the corresponding lactol, which seemed to be unstable on silica gel [3].

Then, the resulting crude (**12**, 9.66 g) was immediately dissolved in THF (150 mL) and *N*-Methoxy-*N*-methyl(triphenylphosphoranylidene)acetamide (**13**, 18.2 g, 50 mmol) was added at 0°C. The reaction mixture was stirred at room temperature for 1.5 hours. After concentration, the residue was directly purified by column chromatography on silica gel (*n*-hexane/EtOAc = 3/1) to afford **7** as white solid (9.8 g, 55% in 4 steps).

White solid; M.p.103–105 °C; ^{1}H NMR (500 MHz, $CDCl_3$): δ 7.35 (dd, *J* = 8.0, 1.4 Hz, 1H), 7.11 (t, *J* = 7.0 Hz, 1H), 7.08 (t, *J* = 7.0 Hz, 1H), 6.76 (t, *J* = 7.9 Hz, 1H), 6.44 (d, *J* = 15.2 Hz, 1H), 5.63 (s,1H), 3.68 (s, 3H), 3.61 (dd, *J* = 6.9, 1.4 Hz, 2H), 3.23 (s, 3H) ppm; ^{13}C NMR (100 MHz, $CDCl_3$): δ 166.7, 150.1, 144.3, 130.3, 129.7, 126.2, 121.5, 120.0, 110.6, 61.8, 33.5, 32.2 ppm; IR (Zn/Se-ATR) 3154, 1657, 1612, 1448 cm^{-1}; HRMS (ESI^+) Cacld. for $C_{12}H_{15}BrNO_3$ ($[M+H]^+$): 300.0230, found: 300.0216; Elemental analysis Cacld. for $C_{12}H_{14}BrNO_3$: C, 48.02; H, 4.70; N, 4.67; found: C, 47.83; H,4.67; N:4.73.

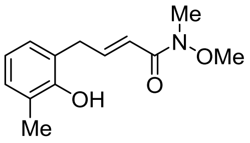

8

(*E*)-4-(2-Hydroxy-3-methylphenyl)-*N*-methoxy-*N*-methylbut-2-enamide (8)

A solution of 2-allyl-6-methylphenol (**10**, 2.01 g, 13.6 mmol) in MeOH (50 mL) was cooled to –78 °C. O_3 gas was bubbled through the solution at –78 °C until the starting material had disappeared. The reaction was quenched with Me_2S (1.1 mL, 14.8 mmol), slowly warmed to room temperature before the addition of water (20 mL). The resulting mixture was stirred for 1 h and concentrated under reduced pressure. The residue was diluted with EtOAc and washed with water. The organic layer was dried over Na_2SO_4 and concentrated in vacuo to give the crude aldehyde, which was similarly, as described above, treated with **13** (4.94 g, 13.6 mmol) in THF (50 mL). The reaction mixture was stirred for 1.5 hours. After concentration, the residue was directly purified by column chromatography on silica gel (*n*-hexane/EtOAc = 3/1) to afford **8** as pale yellow solid (1.53 g, 48% in 2 steps).

Pale yellow solid; M.p.101.8–103.7 °C; ^{1}H NMR (500 MHz, $CDCl_3$): δ 7.14 (dt, J_1 = 15.3 Hz, J_2 = 6.7 Hz, 1H), 7.02 (d, *J* = 7.4 Hz, 1H), 6.97 (t, *J* = 7.4 Hz, 1H), 6.80 (d, *J* = 7.6 Hz, 1H), 6.43 (d, *J* = 15.2 Hz, 1H), 4.88 (s, 1H), 3.66 (s, 3H), 3.57 (dd, J_1 = 6.9 Hz, J_2 = 1.4 Hz, 2H), 3.26 (s, 3H), 2.25 (s, 3H) ppm; ^{13}C NMR (100 MHz, $CDCl_3$): δ 166.9, 152.3, 145.9, 129.4, 128.1, 124.4, 123.9, 120.3, 119.3, 61.7, 33.7, 32.4, 16.1 ppm; IR (Zn/Se-ATR) 3394, 3008, 2977, 2932, 2885, 1653, 1614, 1469, 1196 cm^{-1}; HRMS (ESI^+) Cacld. for $C_{13}H_{17}NNaO_3$ ($[M+Na]^+$): 258.1101, found: 258.1090.

General procedure for asymmetric intramolecular oxa-Michael reaction **(5, 6)**

To a solution of **7** or **8** (1.0 mmol) in CH_2Cl_2 (10 mL) was added catalyst **A**–**E** (1–10 mol %), and the resulting mixture was stirred for the indicated time in Table 1. The reaction mixture was then evaporated and the resulting crude residue was purified by column chromatography on silica gel eluting with *n*-hexane/ethyl acetate (2/1) to afford **5** or **6**.

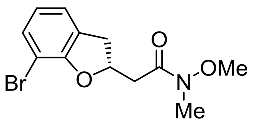

5

(*R*)-2-(7-Bromo-2,3-dihydrobenzofuran-2-yl)-*N*-methoxy-*N*-methylacetamide (5)

White solid; Mp 51–52 °C; ^{1}H NMR (500 MHz, $CDCl_3$): δ 7.24 (d, *J* = 8.0 Hz, 1H), 7.08 (d, *J* = 7.4 Hz, 1H), 6.71 (dd, J_1 = J_2 = 7.6 Hz, 1H), 5.31–5.25 (m, 1H), 3.69 (s, 3H), 3.56 (dd, J_1 = 15.9 Hz, J_2 = 9.0 Hz, 1H), 3.20 (m, 4H), 3.06 (dd, J_1 = 15.8 Hz, J_2 = 7.2 Hz, 1H), 2.78 (dd, J_1 = 16.0 Hz, J_2 = 8.0 Hz, 1H) ppm; ^{13}C NMR (126 MHz, $CDCl_3$): δ 170.9, 156.6, 131.2, 128.3, 124.2, 121.9, 102.6, 80.2, 61.6, 38.4, 36.7, 32.1 ppm; IR (Zn/Se-ATR) 1650, 1453 cm^{-1}; HRMS (ESI$^+$) Cacld.. for $C_{12}H_{15}BrNO_3$ ([M+H]$^+$): 300.0230, found: 300.0206; HPLC [CHIRALPAK IB, *n*-hexane/2-propanol = 95/5, 1.0 mL/min, λ = 220 nm, retention times: (minor) 10.3 min (major) 12.1 min]; $[\alpha]^{26}_D$ +43.6 (*c* 0.83,$CHCl_3$) for 86% ee.

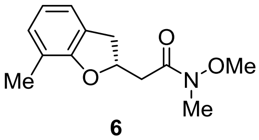

6

(*R*)-*N*-Methoxy-*N*-methyl-2-(7-methyl-2,3-dihydrobenzofuran-2-yl)acetamide (6)

Colorless oil; ^{1}H NMR (400 MHz, $CDCl_3$): δ 6.99 (d, *J* = 7.3 Hz, 1H), 6.92 (d, *J* = 7.3 Hz, 1H), 6.74 (dd, J_1 = J_2 = 7.6 Hz, 1H), 5.23–5.19 (m, 1H), 3.67 (s, 3H), 3.45 (dd, J_1 = 15.6 Hz, J_2 = 9.2 Hz, 1H), 3.21 (s, 3H), 3.13 (dd, J_1 = 15.8 Hz, J_2 = 5.7 Hz, 1H), 2.94 (dd, J_1 = 15.6 Hz, J_2 = 6.9 Hz, 1H), 2.71 (dd, J_1 = 15.6 Hz, J_2 = 7.3 Hz, 1H), 2.19 (s, 3H) ppm; ^{13}C NMR (101 MHz, $CDCl_3$): δ 171.2, 157.4, 129.1, 125.6, 122.3, 120.2, 119.4, 78.8, 61.2, 38.3, 35.9, 31.8, 15.2 ppm; IR (Zn/Se-ATR) 2939, 1660, 1466, 1439, 1193 cm^{-1}; HRMS (ESI$^+$) Cacld. for $C_{13}H_{18}NO_3$ ([M+H]$^+$): 236.1281, found: 236.1284; HPLC [CHIRALPAK IB, *n*-hexane/2-propanol = 95/5, 1.0 mL/min, λ = 238 nm, retention times: (minor) 8.5 min (major) 7.8 min]; $[\alpha]^{26}_D$ +3.88 (*c* 1.48,$CHCl_3$) for 91% ee.

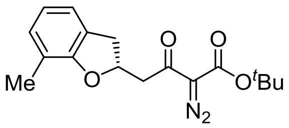

14

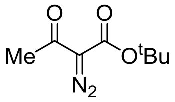

24
(byproduct)

***tert*-Butyl (*R*)-2-diazo-4-(7-methyl-2,3-dihydrobenzofuran-2-yl)-3-oxobutanoate (14)**

To a solution of LHMDS (16.2 mL, 16.2 mmol, 1.0 M solution in THF) in dry THF (16 mL) was added *t*-BuOAc (1.88 g, 16.2 mmol) at −40 ºC dropwise over 10 min under argon atmosphere, and the reaction mixture was stirred at the same temperature for 30 min order to generate lithium enolate. To the reaction mixture was added THF solution (16.0 mL) of **6** (1.15 g, 5.37 mmol) dropwise over 15 min, and the reaction mixture was stirred at the same temperatures until TLC indicated the reaction was complete (30 min). The reaction was quenched with saturated aqueous NH_4Cl solution, and the product was extracted with EtOAc three times. The combined organic layers were washed with brine, dried over Na_2SO_4, and concentrated under reduced pressure to furnish the crude β-ketoester (1.88 g), which was dissolved in THF (40 mL) and CH_3CN (10 mL). To this solution were added 2-azido-1,3-dimethylimidazolinium hexafluorophosphate (ADMP; 2.30 g, 8.06 mmol) and K_2HPO_4 (1.87 g, 10.75 mmol) successively at 0 °C, and the reaction mixture was stirred for 2 h, before being quenched with saturated aqueous NH_4Cl solution (15 mL) and water (15 mL). The organic layers were extracted with $CHCl_3$ three times. The combined extracts were washed with brine (30 mL), dried over anhydrous Na_2SO_4, and concentrated under reduced pressure. The residue was purified by column chromatography on silica gel (*n*-hexane/EtOAc = 10/1) to afford **14** including an inseparable impurity **24** (1.51 g; **14**/**24** = 10/1) as yellow oil.

^{1}H NMR (400 MHz, $CDCl_3$, **14**/**24** = 10:1): product **14**, δ 6.98 (d, J = 7.2 Hz, 1H), 6.91 (d, J = 7.6 Hz, 1H), 6.73 (dd, J_1 = J_2 = 7.6 Hz, 1H), 5.28–5.19 (m, 1H), 3.49 (dd, J_1 = 16.4 Hz, J_2 = 6.4 Hz, 1H), 3.43 (dd, J_1 = 16.0 Hz, J_2 = 6.8 Hz, 1H), 3.11 (dd, J_1 = 16.8 Hz, J_2 = 6.8 Hz, 1H), 2.90 (dd, J_1 = 16.0 Hz, J_2 = 6.4 Hz, 1H) 2.17 (s, 3H), 1.51 (s, 9H); signals of **24**: 2.44 (s, 3H), 1.53 (s, 9H) ppm; ^{13}C NMR (101 MHz, $CDCl_3$): product **14**, δ 190.0, 160.2, 157.5, 129.1, 125.4, 122.2, 120.2, 119.6, 83.3, 78.1, 77.2, 46.3, 35.9, 28.1, 15.1; signal of **24**: 28.2 ppm; HRMS (ESI$^+$) Cacld. for $C_{17}H_{20}N_2NaO_4$ ($[M+Na]^+$): 339.1315, found: 339.1313.

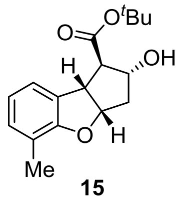

15

***tert*-Butyl (1*R*, 2*R*, 3a*S*, 8b*S*)-2-hydroxy-5-methyl-2,3,3a,8b-tetrahydro-1*H*-cyclopenta-β-benzofuran-1-carboxylate (15)**

To a crude of **14** (758.0 mg, 2.40 mmol) in CH_2Cl_2 (20 mL) was added $Rh_2(OAc)_4$ (10.6 mg, 1.0 mol %), and the resulting mixture was stirred at ambient temperature for 15 min, when TLC indicated the reaction

was complete. MeOH (10 mL) was then added, and $NaBH_4$ (271.9 mg, 7.19 mmol) was added portionwise –40 °C. The mixture was stirred at –40 °C for 2 h and at the ambient temperature for additional 2 h, before being quenched with saturated aqueous NH_4Cl solution (20 mL). The organic materials were extracted with EtOAc three times, and the combined organic layer was washed with brine (50 mL), dried over anhydrous Na_2SO_4, and evaporated to give the crude residue, which was purified by column chromatography on silica gel eluting with *n*-hexane/ethyl acetate (7/1) to afford **15** (490.0 mg, 60% in 4 steps from **6**) as white solid; Mp.73.2–73.4 °C; ^{1}H NMR (500 MHz, $CDCl_3$): δ 7.12 (d, J = 7.2 Hz, 1H), 6.97 (d, J = 7.4 Hz, 1H), 6.79 (dd, J_1 = J_2 = 7.4 Hz, 1H), 5.17 (ddd, J_1 = 9.0 Hz, J_2 = 7.2 Hz, J_3 = 5.2 Hz, 1H), 4.38–4.33 (m, 1H), 3.92 (t, J = 8.0 Hz, 1H), 2.79 (t, J = 7.0 Hz, 1H), 2.59 (ddd, J_1 = 13.4 Hz, J_2 = J_3 = 6.7 Hz, 1H), 2.39 (dd, J_1 = 4.7 Hz, J_2 = 3.0 Hz, 1H), 2.20 (s, 3H), 2.07 (ddd, J_1 = 13.4 Hz, J_2 = 8.0 Hz, J_3 = 5.3 Hz, 1H), 1.53 (s, 9H) ppm; ^{13}C NMR (126 MHz, $CDCl_3$): δ 172.7, 157.3, 129.9, 129.0, 122.2, 120.7, 120.0, 84.8, 81.8, 74.6, 60.3, 48.0, 41.1, 28.2, 15.3 ppm; IR (Zn/Se-ATR) 3466, 2978, 2872, 1699 cm^{-1}; HRMS (ESI$^+$) Cacld. for $C_{17}H_{22}NaO_4$ ([M+Na]$^+$): 313.1410, Found: 313.1398; $[\alpha]^{26}_D$ +80.2 (*c* 0.79, $CHCl_3$).

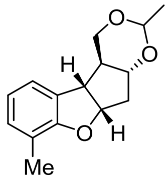

16

Acetal 16

To a solution of **15** (513.5 mg, 1.77 mmol) in Et_2O (17 mL) was added $LiBH_4$ (115.6 mg, 5.31 mol), and the resulting mixture was stirred at ambient temperature for 9 h. The reaction was quenched with 1 N aqueous HCl solution (10 mL), and the organic materials were extracted with EtOAc three times. The combined extracts were washed with brine (20 mL), dried over anhydrous Na_2SO_4, and evaporated to afford the crude diol (367.9 mg), which was then dissolved in THF (16 mL). To this solution were added 1,1-diethoxyethane (2.5 mL, 17.6 mmol) and *p*-TsOH·H_2O (31.8 mg, 10 mol%), and the reaction mixture was stirred at 60 °C for 3 h before being quenched with saturated aqueous $NaHCO_3$ solution (15 mL). The mixture was extracted with EtOAc three times, and the combined extracts were washed with brine (30 mL), dried over anhydrous Na_2SO_4, and evaporated to give the crude acetal, which was purified by column chromatography on silica gel eluting with *n*-hexane/ethyl acetate (15/1) to afford **16** (292.6 mg, 71% in 2 steps from **15**) as white solid; Mp.125.5–129.0 °C; ^{1}H NMR (500 MHz, $CDCl_3$): δ 6.97 (d, J = 7.4 Hz, 1H), 6.91 (d, J = 7.2 Hz, 1H), 6.75 (dd, J_1 = J_2 = 7.4 Hz, 1H), 5.10 (q, J = 7.5 Hz, 1H), 4.72 (q, J = 5.0 Hz, 1H), 4.39 (dd, J_1 = 10.5 Hz, J_2 = 4.2 Hz, 1H), 3.71 (dd, J_1 = J_2 = 10.5 Hz, 1H), 3.41 (td, J_1 = 11.0, J_2 = 6.3 Hz, 1H), 3.15 (t, J = 10.0 Hz, 1H), 2.74 (ddd, J_1 = 12.8 Hz, J_2 = J_3 = 6.4 Hz, 1H), 2.20 (s, 3H), 1.97 (td, J_1 = 12.1 Hz, J_2 = 5.9 Hz, 1H), 1.88 (ddd, J_1 = 21.6 Hz, J_2 = 10.7 Hz, J_3 = 4.2 Hz, 1H), 1.36 (d, J = 5.2 Hz, 3H) ppm; ^{13}C NMR (126 MHz, $CDCl_3$): δ 157.5, 129.8, 128.0, 121.3, 120.3, 120.1, 99.5, 83.4, 79.6, 70.8, 48.1, 45.3, 37.9, 20.6, 15.3 ppm; IR (Zn/Se-ATR) 2952, 1464, 1384 cm^{-1}; $[\alpha]^{26}_D$ +7.5 (*c* 0.4, $CHCl_3$).

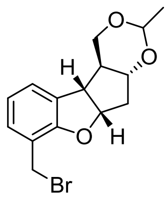

17

Bromide 17

To the solution of **16** (40.0 mg, 0.162 mmol) and NBS (34.7 mg, 0.195 mmol) in CCl_4 (2.5 mL) was added azobisisobutyronitrile (AIBN, 3.2 mg, 0.0195 mmol) at room temperature, and the mixture was heated under reflux for 3 h. The crude mixture was filtered through a pad of Celite, and the filtrate was concentrated to give the crude, which was then purified by column chromatography on silica gel eluting with *n*-hexane/ethyl acetate (10/1) to afford **17** (7.4 mg, 14%) as yellow oil; ^{1}H NMR (500 MHz, $CDCl_3$): δ 7.17 (d, J = 7.5 Hz, 1H), 7.04 (d, J = 7.0 Hz, 1H), 6.82 (dd, J_1 = J_2 = 7.5 Hz, 1H), 5.23–5.19 (m, 1H), 4.73 (q, J = 5.0 Hz, 1H), 4.53 (d, J = 10.0 Hz, 1H), 4.46 (d, J = 10.0 Hz, 1H), 4.40 (dd, J_1 = 10.0 Hz, J_2 = 5.0 Hz, 1H), 3.73 (t, J_1 = 11.0 Hz, 1H), 3.44 (td, J_1 = 11.0 Hz, J_2 = 6.5 Hz, 1H), 3.18 (dd, J_1 = 11.0 Hz, J_2 = 9.0 Hz, 1H), 2.78 (ddd, J_1 = 12.5 Hz, J_2 = J_3 = 6.5 Hz, 1H), 2.01 (td, J_1 = 12.2 Hz, J_2 = 5.8 Hz, 1H), 1.85 (ddd, J_1 = 21.7 Hz, J_2 = 10.8 Hz, J_3 = 4.4 Hz, 1H), 1.36 (d, J = 5.2 Hz, 3H) ppm; ^{13}C NMR (126 MHz, $CDCl_3$): δ 157.3, 129.6, 129.5, 124.5, 120.9, 99.6, 84.5, 79.5, 70.7, 48.2, 45.1, 37.8, 28.0, 20.6 ppm; IR (Zn/Se-ATR) 2986, 2938, 2856, 1728, 1599, 1455 cm^{-1}; $[\alpha]^{26}_{D}$ +40.6 (*c* 1.15, $CHCl_3$).

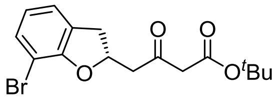

19

***tert*-Butyl (*R*)-4-(7-bromo-2,3-dihydrobenzofuran-2-yl)-3-oxobutanoate (19)**

To a solution of LiHMDS (1.2 mL, 1.2 mmol, 1.0 M solution in THF) in dry THF (1.2 mL) was added *t*-BuOAc (139.4 mg, 1.2 mmol) at –40 ºC dropwise over 10 min under argon atmosphere, and the reaction mixture was stirred at the same temperature for 30 min. To the reaction mixture was added $AlBr_3$ (324.9 mg, 1.22 mmol) in THF (3.6 mL) dropwise over 10 min, and stirred at the same temperature for 20 min, before the subsequent dropwise addition of amide **5** (120.1 mg, 0.40 mmol) in THF (1.0 mL) over 15 min. The reaction mixture was stirred at the same temperatures until TLC indicated the reaction was complete (2 h), and then quenched with saturated aqueous NH_4Cl solution. The product was extracted with EtOAc three times, and the combined organic layers were washed with brine, dried over Na_2SO_4, and concentrated under reduced pressure. The residue was purified by column chromatography on silica gel (*n*-hexane/EtOAc = 10/1) to afford **28** (128.1 mg, 90%) as a colorless oil.; ^{1}H NMR (500 MHz, $CDCl_3$, keto/enol = 11:1); keto form, δ 7.26 (d, J = 6.3 Hz, 1H), 7.08 (d, J = 7.2 Hz, 1H), 6.73 (dd, J_1 = J_2 = 7.7 Hz, 1H), 5.24 (dt, J_1 = 15.0 Hz, J_2 = 7.5 Hz, 1H), 3.56 (dd, J_1 = 15.9 Hz, J_2 = 9.0 Hz, 1H), 3.44 (s, 2H), 3.24 (dd, J_1 = 17.2 Hz, J_2 = 5.4 Hz, 1H), 2.97 (dd, J_1 = 16.9 Hz, J_2 = 7.4 Hz, 2H), 1.47 (s, 9H); selected signals for enol form: 5.19–5.13 (m, 1H), 5.00 (s, 1H), 3.10 (dd, J_1 = 15.8 Hz, J_2 = 6.6 Hz, 1H), 2.83 (dd, J_1 = 14.0 Hz, J_2 = 6.0 Hz,

1H), 2.48 (dd, J_1 = 14.2 Hz, J_2 = 8.2 Hz, 1H), 1.50 (s, 9H) ppm; ^{13}C NMR (100 MHz, $CDCl_3$): δ 200.6, 166.0, 156.3, 131.2, 127.7, 123.9, 122.0, 102.6, 82.3, 78.9, 51.0, 48.5, 36.4, 27.9 ppm; IR (Zn/Se-ATR) 2979, 1733, 1713, 1454 cm^{-1}; HRMS (ESI^+) Cacld. for $C_{16}H_{19}NaO_4Br$ ($[M+Na]^+$): 377.0359, found: 377.0346; $[\alpha]^{26}_D$ +5.2 (*c* 1.85, $CHCl_3$).

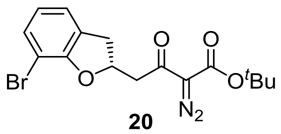

20

***tert*-Butyl (*R*)-4-(7-bromo-2,3-dihydrobenzofuran-2-yl)-2-diazo-3-oxobutanoate (20)**

To a mixture of ketoester **19** (825.2 mg, 2.32 mmol) and 2-azido-1,3-dimethylimidazolinium hexafluorophosphate (ADMP; 794.8 mg, 2.79 mmol) in THF/CH_3CN (16 mL/4.0 mL) was added K_2HPO_4 (809.3 mg, 4.65 mmol) at 0 °C, and the reaction mixture was stirred at the same temperature for 2 h. The reaction was quenched with saturated aqueous NH_4Cl solution (10 mL) and additional water (8 mL). The organic materials were extracted with $CHCl_3$ three times, and the combined extracts were washed with brine (20 mL), dried over anhydrous Na_2SO_4, and concentrated under reduce pressure to afford the crude diazoester **20**, which was then purified by column chromatography on silica gel eluting with *n*-hexane/ethyl acetate (8/1) to afford white solid. This solid was recrystallized with hexane/Et_2O (1:1), and the enantioenriched product **20** was obtained from filtrate (376.6 mg, 43%, 95% ee). White solid; Mp. 88.6–92.9 °C; ^{1}H NMR (400 MHz, $CDCl_3$): δ 7.24 (d, *J* = 8.2 Hz, 1H), 7.07 (d, *J* = 8.2 Hz, 1H), 6.71 (dd, J_1 = J_2 = 7.8 Hz, 1H), 5.36–5.29 (m, 1H), 3.57–3.51 (m, 2H), 3.17 (dd, J_1 = 17.2 Hz, J_2 = 7.1 Hz, 1H), 3.00 (dd, J_1 = 15.8 Hz, J_2 = 7.1 Hz, 1H), 1.52 (s, 9H) ppm; ^{13}C NMR (100 MHz, $CDCl_3$): δ 189.7, 160.3, 156.5, 131.1, 127.8, 123.9, 121.8, 102.7, 83.5, 79.3, 77.2, 46.0, 36.5, 28.3 ppm; IR (Zn/Se-ATR) 2130, 1710, 1643 cm^{-1}; HRMS (ESI^+) Cacld. for $C_{16}H_{17}N_2NaO_4$ ($[M+Na]^+$): 403.0269, Found: 403.0264; HPLC [Chiralpak IB, *n*-hexane/2-propanol = 99/1, 1.0 mL/min, λ = 254 nm, retention times: (major) 16.7 min (minor) 10.1 min]; $[\alpha]^{26}_D$ +24.3 (*c* 0.86,$CHCl_3$) for 95% ee.

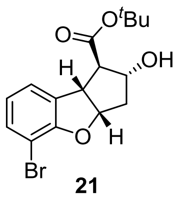

21

***tert*-Butyl (1*R*,2*R*,3a*S*,8b*S*)-5-bromo-2-hydroxy-2,3,3*a*,8*b*-tetrahydro-1*H*-cyclopenta-β-benzofuran-1-carboxylate (21)**

To a solution of **20** (376.0 mg, 0.986 mmol) in CH_2Cl_2 (10 mL) was added $Rh_2(OAc)_4$ (4.4 mg, 1.0 mol%), and the resulting mixture was stirred at ambient temperature for 15 min, when TLC indicated the reaction was complete. MeOH (5.0 mL) was added, and $NaBH_4$ (111.9 mg, 2.96 mmol) was added portionwise at –40 °C. The mixture was stirred for 4 h before being quenched with saturated aqueous NH_4Cl solution (10

mL). The organic materials were extracted with EtOAc three times, and the combined organic layer was washed with brine (20 mL), dried over anhydrous Na_2SO_4, and concentrated under reduce pressure to afford the crude residue, which was purified by column chromatography on silica gel eluting with *n*-hexane/ethyl acetate (6/1) to afford **21** (235.0 mg, 67% in 2 steps from **20**) as white solid. Mp.109.0–112.0 °C; ^{1}H NMR (400 MHz, $CDCl_3$): δ 7.30 (d, J = 8.1 Hz, 1H), 7.21 (d, J = 7.0 Hz, 1H), 6.76 (dd, J_1 = J_2 = 7.8 Hz, 1H), 5.29–5.24 (m, 1H), 4.38 (dd, J_1 = 13.9 Hz, J_2 = 7.0 Hz, 1H), 4.02 (dd, J_1 = J_2 = 8.1 Hz, 1H), 2.80 (dd, J_1 = J_2 = 7.0 Hz, 1H), 2.38 (ddd, J_1 = 13.8 Hz, J_2 = J_3 = 7.0 Hz, 1H), 2.33 (brs, 1H), 2.14 (ddd, J_1 = 13.9 Hz, J_2 = 8.1 Hz, J_3 = 5.2 Hz, 1 H), 2.33 (s, 9H) ppm; ^{13}C NMR (100 MHz, $CDCl_3$): δ 172.2, 156.3, 131.8, 131.2, 123.8, 122.1, 102.7, 85.7, 82.0, 74.4, 48.5, 41.0, 28.1 ppm; IR (Zn/Se-ATR) 3477, 2982, 2899, 1696, 1459 cm^{-1}; HRMS (ESI$^+$) Cacld. for $C_{16}H_{19}BrNaO_4$ ([M+Na]$^+$): 377.0359, Found: 377.0366; HPLC [Chiralpak OJ3, *n*-hexane/2-propanol = 95/5, 1.0 mL/min, λ = 254 nm, retention times: (major) 11.7 min (minor) 14.9 min]; $[\alpha]^{26}_D$ +82.7 (*c* 0.70, $CHCl_3$).

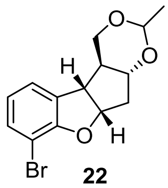

22

Acetal (22)

To a solution of **21** (467.7 mg, 1.32 mmol) in Et_2O (10 mL) was added $LiBH_4$ (86.03 mg, 3.95 mol), and the resulting mixture was stirred at ambient temperature for 15 h. The reaction was quenched with 1 N aqueous HCl solution (20 mL), and the organic materials were extracted with EtOAc three times. The combined extracts were washed with brine (20 mL), dried over anhydrous Na_2SO_4, and concentrated under reduced pressure to afford the crude diol, which was then dissolved in THF (10 mL). To this solution, were added 1,1-diethoxyethane (1.5 mL, 10.5 mmol) and *p*-TsOH·H_2O (25.2 mg, 10 mol %), and the reaction mixture was stirred at 60 °C for 3 h, before being quenched with saturated aqueous $NaHCO_3$ solution (10 mL). The product was extracted with EtOAc three times, and the combined extracts were washed with brine (10 mL), dried over anhydrous Na_2SO_4, and evaporated to give the resulting crude residue, which was purified by column chromatography on silica gel eluting with *n*-hexane/ethyl acetate (8/1) to afford **22** (283.2 mg, 69% in 2 steps from **21**) as colorless oil. ^{1}H NMR (400 MHz, $CDCl_3$): δ 7.31 (d, J = 8.2 Hz, 1H), 7.02 (d, J = 7.3 Hz, 1H), 6.74 (dd, J_1 = J_2 = 7.6 Hz, 1H), 5.22 (td, J_1 = 7.9 Hz, J_2 = 7.5 Hz, 1H), 4.73 (q, J = 5.0 Hz, 1H), 4.39 (dd, J_1 = 10.5 Hz, J_2 = 4.6 Hz, 1H), 3.73 (dd, J_1 = J_2 = 10.5 Hz, 1H), 3.42 (td, J_1 = 11.2, J_2 = 6.1 Hz, 1H), 3.26 (dd, J_1 = 11.0 Hz, J_2 = 9.2 Hz, 1H), 2.78 (ddd, J_1 = 13.0 Hz, J_2 = J_3 = 6.5 Hz, 1H), 2.06 (td, J_1 = 12.1 Hz, J_2 = 6.0 Hz, 1H), 1.88 (ddd, J_1 = 12.9 Hz, J_2 = 7.5 Hz, J_3 = 5.2 Hz, 1H), 1.36 (d, J = 5.0 Hz, 3H) ppm; ^{13}C NMR (126 MHz, $CDCl_3$): δ 156.4, 131.8, 130.2, 123.0, 122.0, 102.9, 99.6, 84.4, 79.4, 70.6, 48.1, 46.0, 37.7, 20.6 ppm; IR (Zn/Se-ATR) 2990, 1451, 1388 cm^{-1}; HRMS (ESI$^+$) Cacld. for $C_{12}H_{13}NaO_3Br$ ([M-C_2H_2+Na]$^+$): 306.9940, Found: 306.9925; $[\alpha]^{26}_D$ +43.0 (*c* 0.98, $CHCl_3$)..

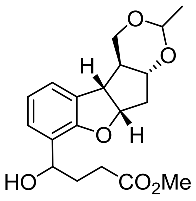

23

Ester 23

To a solution of the acetal **22** (76.7 mg, 0.246 mmol) in THF (2.5 mL) was added TMEDA (0.04 mL 0.32 mmol) at –78 °C, and the reaction mixture was stirred for 10 minutes. To this solution was added a *n*-hexane solution of *n*-BuLi (1.55 M, 0.2 mL, 0.3 mmol) at –78 °C, and the mixture stirred at the same temperature for 5 minutes before the subsequent dropwise addition of 3-formylpropionate (85.7 mg, 0.738 mmol) in THF (1.0 mL) over 5 minutes. The reaction mixture was stirred at –78 °C for 30 min, and quenched with saturated aqueous NH_4Cl solution (3.0 mL). The organic materials were extracted with EtOAc three times, and the combined extracts were washed with brine (5 mL), dried over anhydrous Na_2SO_4, and concentrated under reduced pressure to give crude ester. The residue was purified by column chromatography on silica gel eluting with *n*-hexane/ethyl acetate (2/1) to afford **23** (49.8 mg, 58%) as colorless oil. ^{1}H NMR (400 MHz, $CDCl_3$): δ 7.15 (d, J = 7.8 Hz, 1H), 7.06 (d, J = 7.3 Hz, 1H), 6.74 (t, J = 7.3 Hz, 1H), 5.16 (q, J = 7.2 Hz, 1H), 4.80 (q, J = 6.0 Hz, 1H), 4.73 (q, J = 4.9 Hz, 1H), 4.39 (dd, J_1 = 10.5 Hz, J_2 = 4.1 Hz, 1H), 3.73 (t, J = 10.8 Hz, 1H), 3.67 (s, 3H), 3.43 (td, J_1 = 11.2, J_2 = 6.4 Hz, 1H), 3.16 (t, J = 9.8 Hz, 1H), 2.78–2.70 (m, 2H), 2.45 (q, J = 6.8 Hz, 2H), 2.11 (ddd, J_1 = 13.6 Hz, J_2 = J_3 = 6.8 Hz, 1H), 1.96 (td, J_1 = 12.1 Hz, J_2 = 5.8 Hz, 1H), 1.82 (qd, J_1 = 10.8 Hz, J_2 = 4.1 Hz, 1H), 1.69 (d, J = 6.8 Hz, 1H), 1.36 (d, J = 4.8 Hz, 3H) ppm; ^{13}C NMR (101 MHz, $CDCl_3$): δ 174.3, 155.7, 129.0, 126.2, 125.9, 123.1, 120.9, 99.6, 84.3, 79.5, 70.7, 70.2, 51.6, 48.2, 44.8, 37.9, 32.1, 30.4, 20.6 ppm; IR (Zn/Se-ATR) 3461, 2949, 1732, 1450 cm^{-1}; HRMS (ESI$^+$) Cacld. for $C_{19}H_{24}NaO_6$ ($[M+Na]^+$): 371.1465, Found: 371.1477; $[\alpha]^{26}_D$ +31.4 (c 0.67, $CHCl_3$).

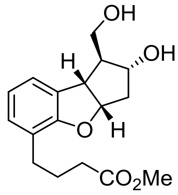

2

Methyl 4-((1*S*,2*R*,3a*S*,8b*S*)-2-Hydroxy-1-(hydroxymethyl)-2,3,3a,8b-tetrahydro-1*H*-cyclopenta-β-benzofuran-5-yl)butanoate (2) [4]

To the solution of **23** (103.5 mg 0.297 mmol) in wet MeOH (5.0 mL) was added pyridinium *p*-toluenesulfonate (PPTS, 630.0 mg 2.51 mmol) at 0 °C, and the reaction mixture was stirred at room temperature for 3 h. The organic materials were extracted with EtOAc three times, and the combined extracts were washed with brine (5 mL), dried over anhydrous Na_2SO_4, and evaporated to give the crude diol, which was then dissolved in CH_2Cl_2 (4.0 mL). To this solution were added Et_3SiH (0.5 mL, 3.13

mmol) and TFA (0.1 mL 1.35 mmol) at 0 °C dropwise, and the reaction mixture was stirred at 0 °C for 1.5 h, before being quenched with saturated aqueous $NaHCO_3$ solution (3.0 mL). The organic materials were extracted with EtOAc three times, and the combined extracts were washed with brine, dried over anhydrous Na_2SO_4, and concentrated under reduced pressure to afford crude **2**, which was then purified by preparative thin layer chromatography on silica gel with *n*-hexane/ethyl acetate (1/4) as eluent to afford key intermediate **2** (33.7 mg 37% in 2 steps from **23**) as white solid. Mp.51.6–55.6 °C; ^1H NMR (400 MHz, $CDCl_3$): δ 7.02 (d, J = 7.3 Hz, 1H), 6.95 (d, J = 7.3 Hz, 1H), 6.79 (t, J = 7.3 Hz, 1H), 5.13 (ddd, J_1 = 8.8 Hz, J_2 = 7.2 Hz, J_3 = 4.8 Hz, 1H), 4.13 (q, J = 6.9 Hz, 1H), 3.97 (dd, J_1 = 10.3 Hz, J_2 = 5.3 Hz, 1H), 3.79 (t, J = 9.2 Hz, 1H), 3.65 (s, 3H), 3.42 (t, J = 8.0 Hz, 1H), 2.55–2.65 (m, 3H), 2.32 (t, J = 7.3 Hz, 2H), 2.17 (ddd, J_1 = 13.4 Hz, J_2 = J_3 = 6.7 Hz, 1H), 2.05 (ddd, J_1 = 13.2 Hz, J_2 = 8.0 Hz, J_3 = *5.2* Hz, 1H), 1.90–2.01 (m, 2H), 1.58–1.62 (brs, 2H) ppm; ^{13}C NMR (101 MHz, $CDCl_3$): δ 174.2, 157.1, 130.2, 128.9, 123.4, 122.0, 120.7, 85.4, 75.9, 64.9, 56.8, 51.5, 47.5, 41.9, 33.4, 29.2, 24.8 ppm; IR (Zn/Se-ATR) 3383, 2929, 1728, 1453 cm^{-1}; HRMS (ESI^+) Cacld. for $C_{17}H_{22}NaO_5$ ($[M+Na]^+$): 329.1359, Found: 329.1367; HPLC [Chiralpak AD, *n*-hexane/2-propanol = 95/5, 1.0 mL/min, λ = 238 nm, retention times: (major) 42.8 min (minor) 38.3 min]; $[\alpha]^{26}_D$ +12.5 (*c* 1.25, $CHCl_3$) for 95% ee.

3. Reference

(1) Deping, C.; Kevin, D. J.; Duke, F. M.; Antony, S. N.; Rosanna, T.; Kenneth, W. J.; Michael, Z. N. *Eur. Pat. Appl.* W52313, 2004.

(2) Mitchell, J. M.; Finney, N. S. *Tetrahedron Lett*. **2000**, *41*, 8431.

(3) Hintermann, L.; Ackerstaff, J.; Boeck, F. *Chem. Eur. J.* **2013**, *19*, 2311

(4) Reddy, N. K.; Vijaykumar, B. V. D.; Chandrasekhar, S. *Org. Lett*. **2012**, *14*, 299.

4. Copies of ^{1}H and ^{13}C NMR charts

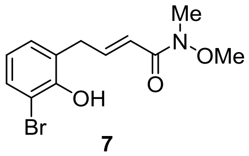

7

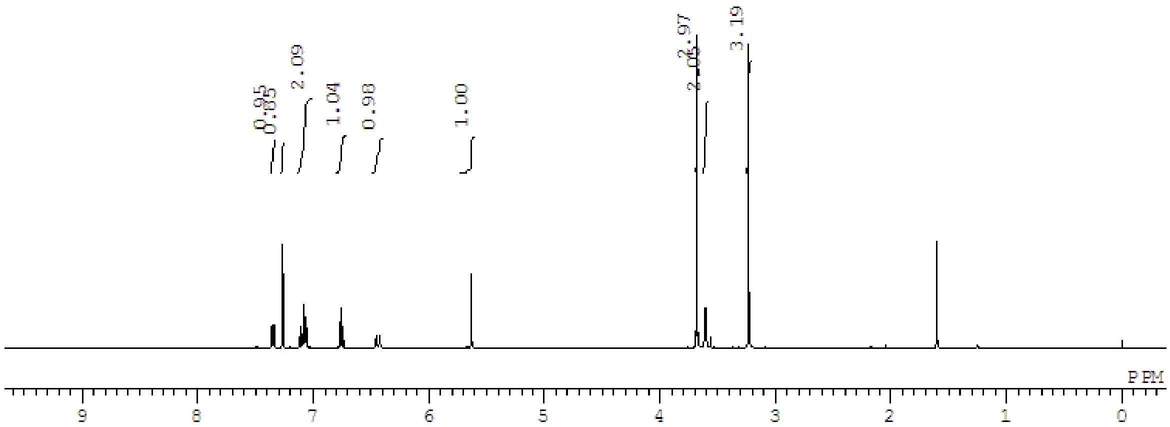

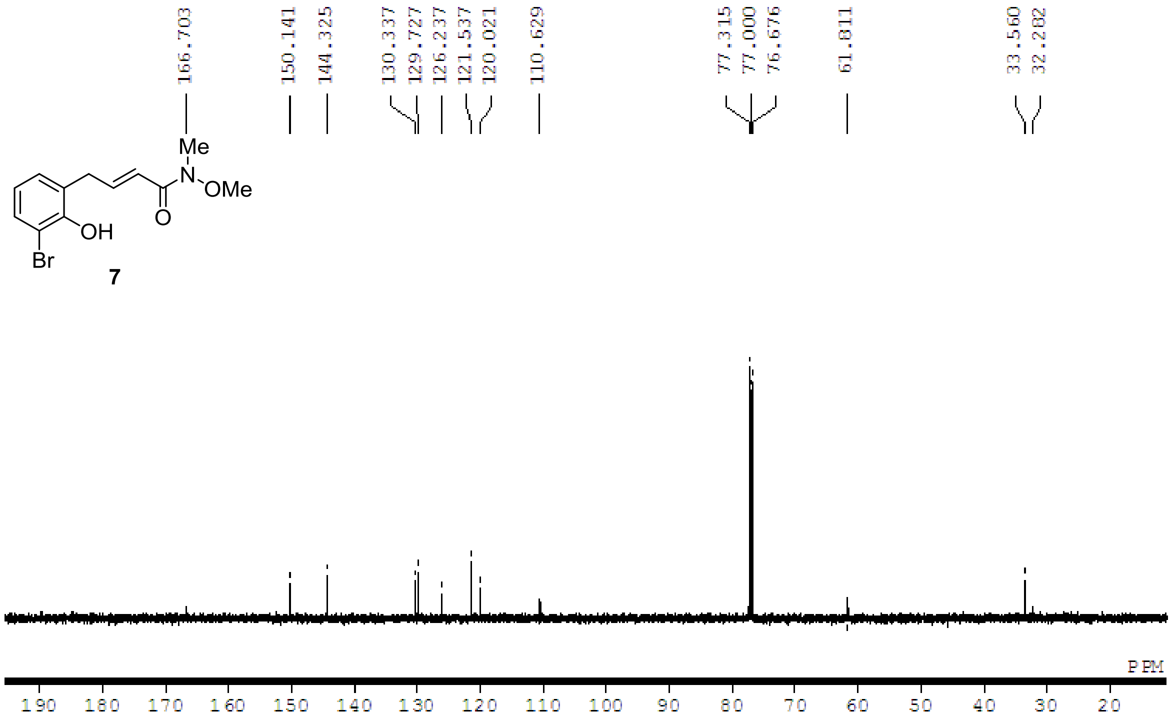

7

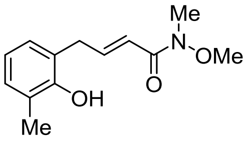
Me
N
OMe
O
OH
Me

8

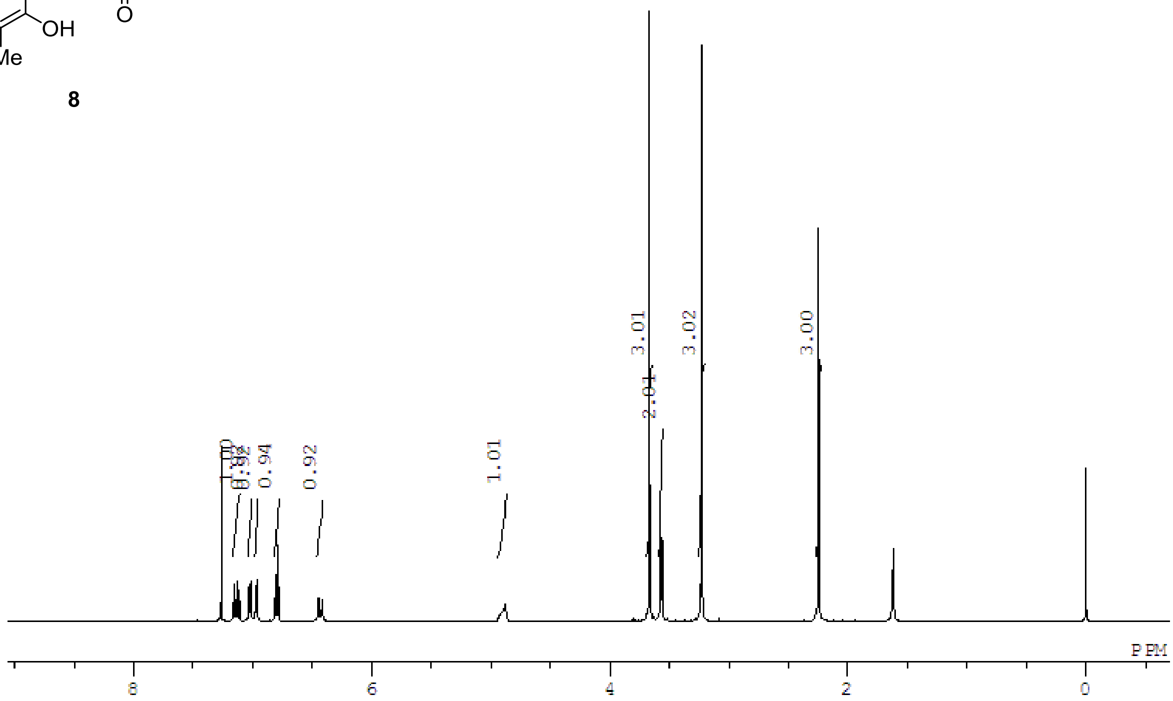
3.01
3.02
3.00
2.01
0.94
0.92
1.01
PPM
8
6
4
2
0

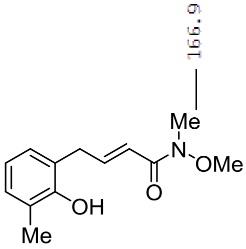
Me
N
OMe
O
OH
Me

8

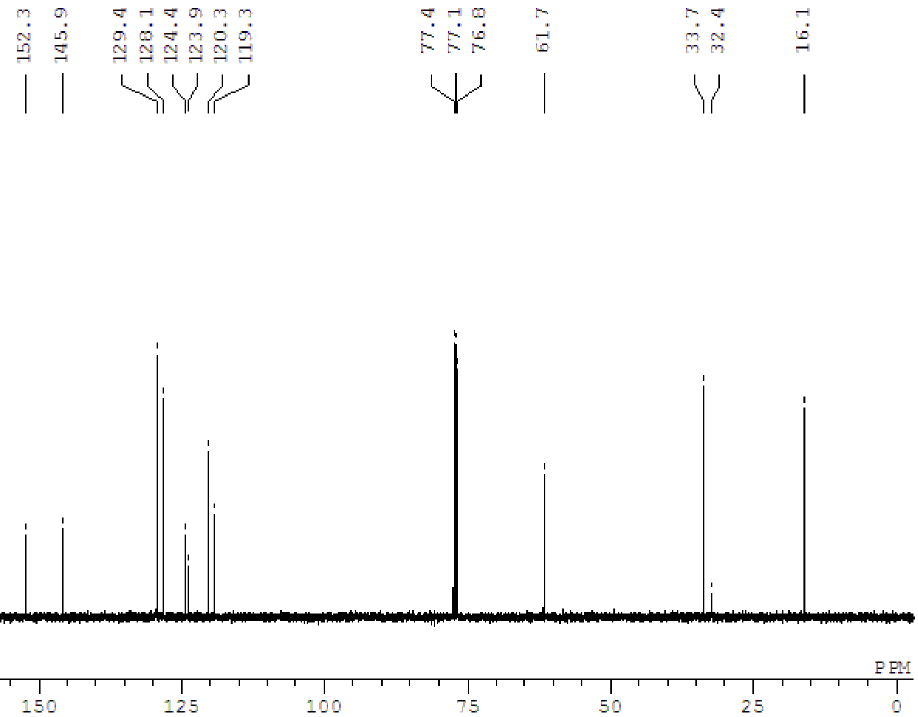
166.9
152.3
145.9
129.4
128.1
124.4
123.9
120.3
119.3
77.4
77.1
76.8
61.7
33.7
32.4
16.1
PPM
200
175
150
125
100
75
50
25
0

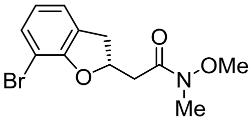
Br
O
O
N
OMe
Me

5

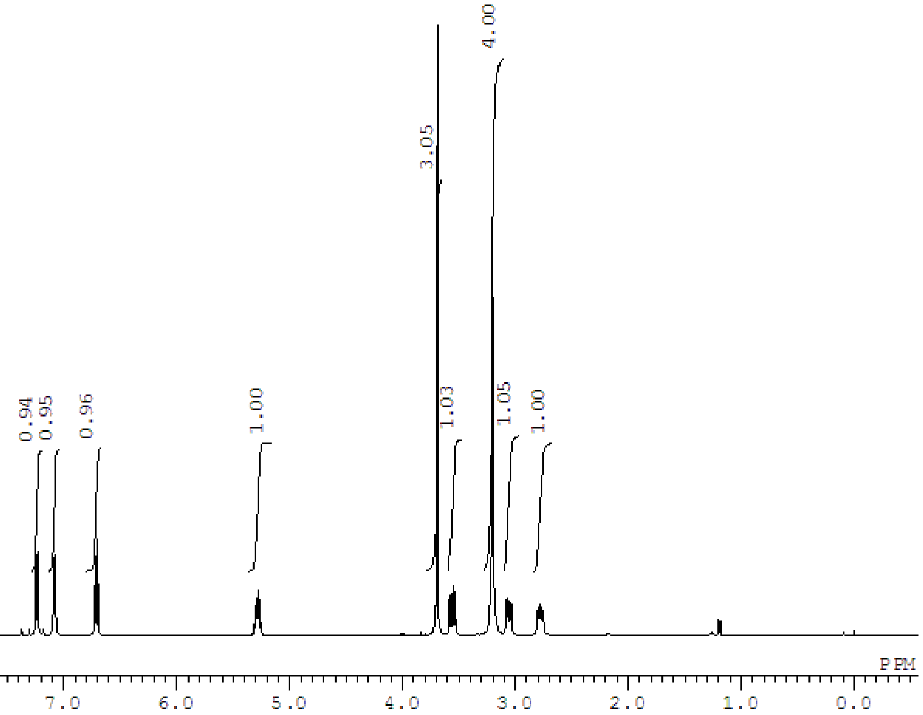
0.94
0.95
0.96
1.00
3.05
1.03
4.00
1.05
1.00
PPM
9.0 8.0 7.0 6.0 5.0 4.0 3.0 2.0 1.0 0.0

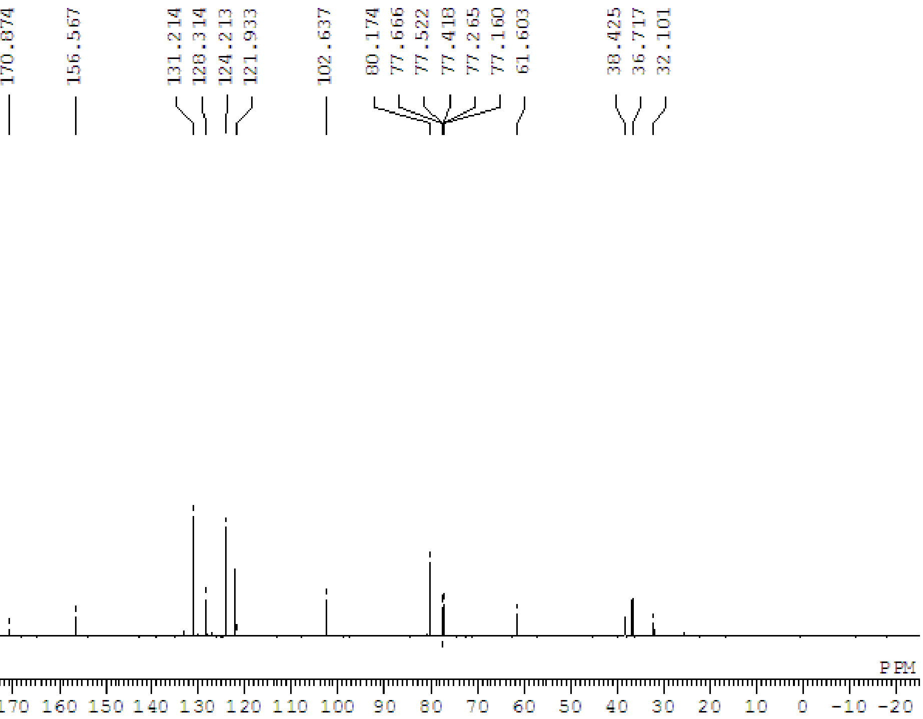
170.874
156.567
131.214
128.314
124.213
121.933
102.637
80.174
77.666
77.522
77.418
77.265
77.160
61.603
38.425
36.717
32.101
PPM
220 210 200 190 180 170 160 150 140 130 120 110 100 90 80 70 60 50 40 30 20 10 0 -10 -20

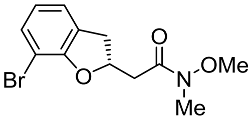
Br
O
O
N
OMe
Me

5

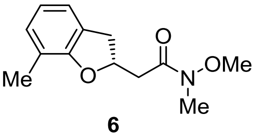
O
OMe
N
Me
Me
O

6

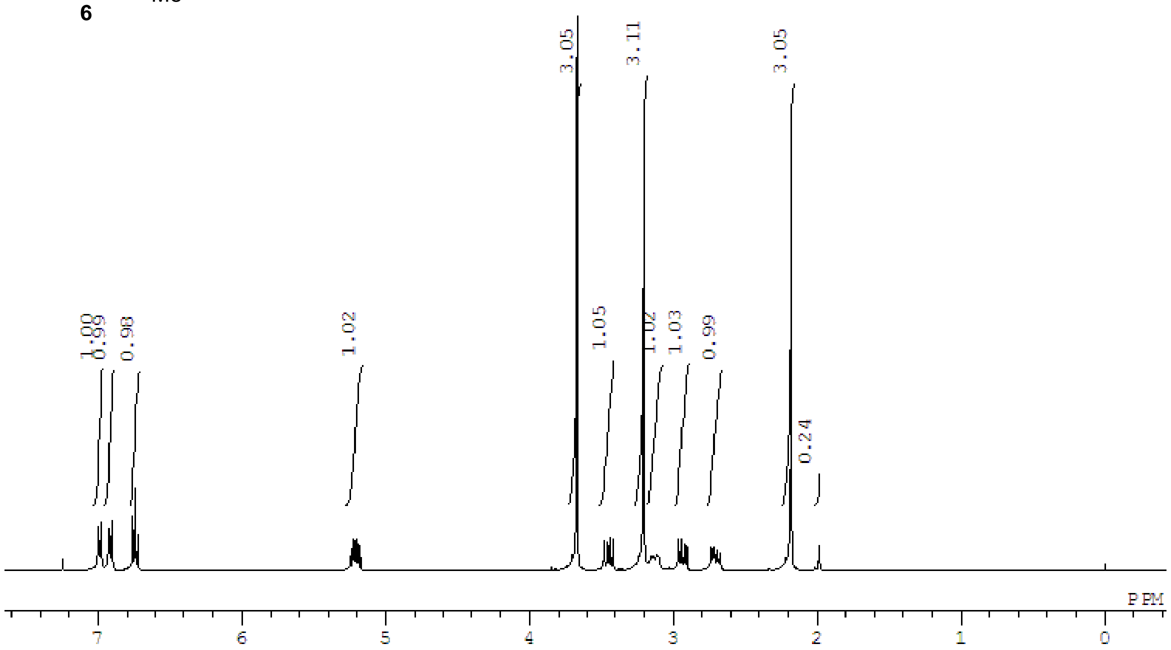
3.05
3.11
3.05
1.00
0.99
0.98
1.02
1.05
1.02
1.03
0.99
0.24
PPM
7
6
5
4
3
2
1
0

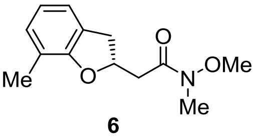
O
OMe
N
Me
Me
O

6

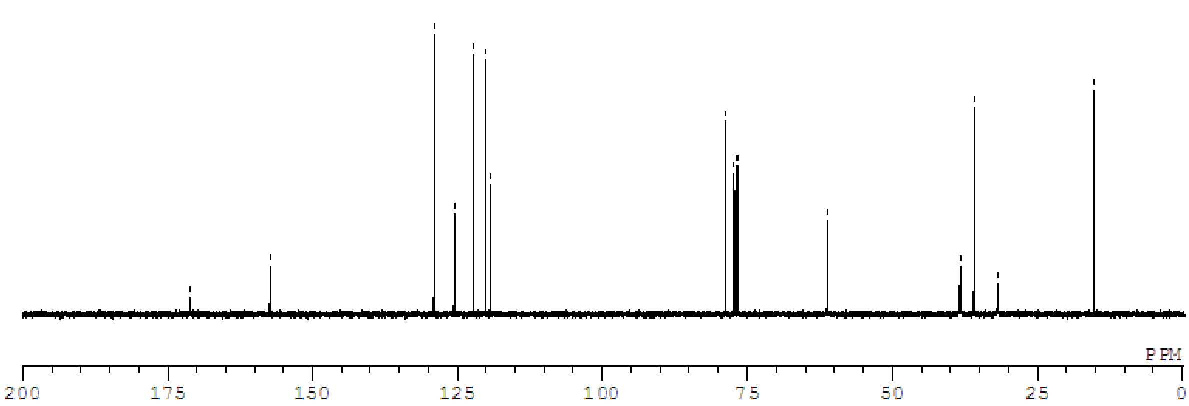
171.2
157.4
129.1
125.6
122.3
120.2
119.4
78.8
77.3
77.0
76.7
61.2
38.3
35.9
31.8
15.2
PPM
200
175
150
125
100
75
50
25
0

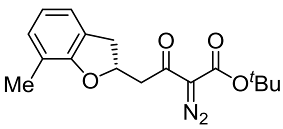

14

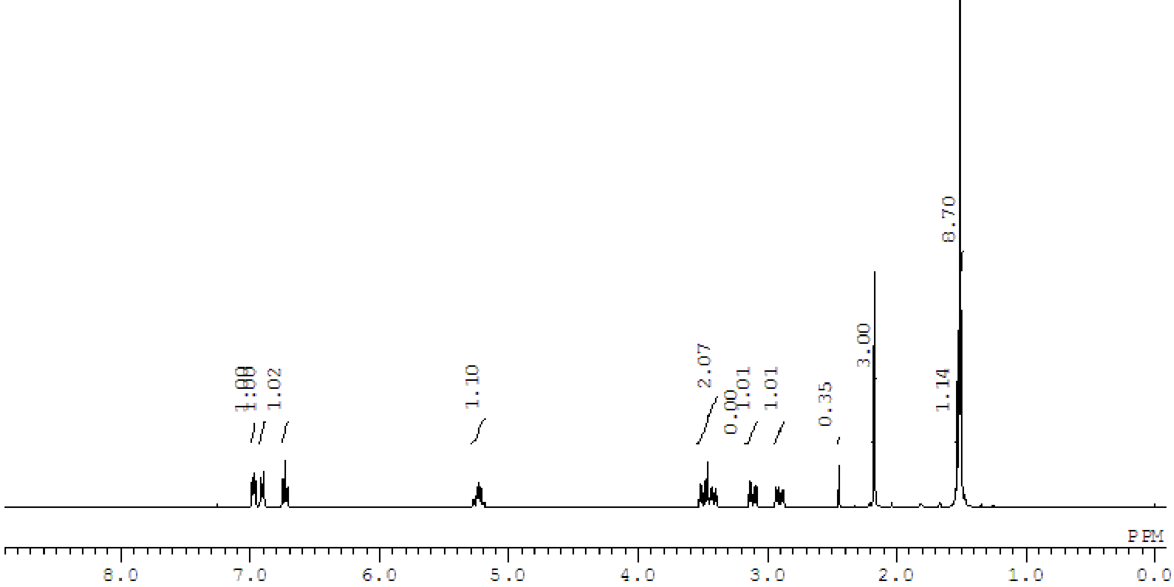

1.00
1.00
1.02
1.10
2.07
0.00
1.01
1.01
0.35
3.00
1.14
8.70
PPM
8.0
7.0
6.0
5.0
4.0
3.0
2.0
1.0
0.0

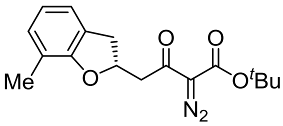

14

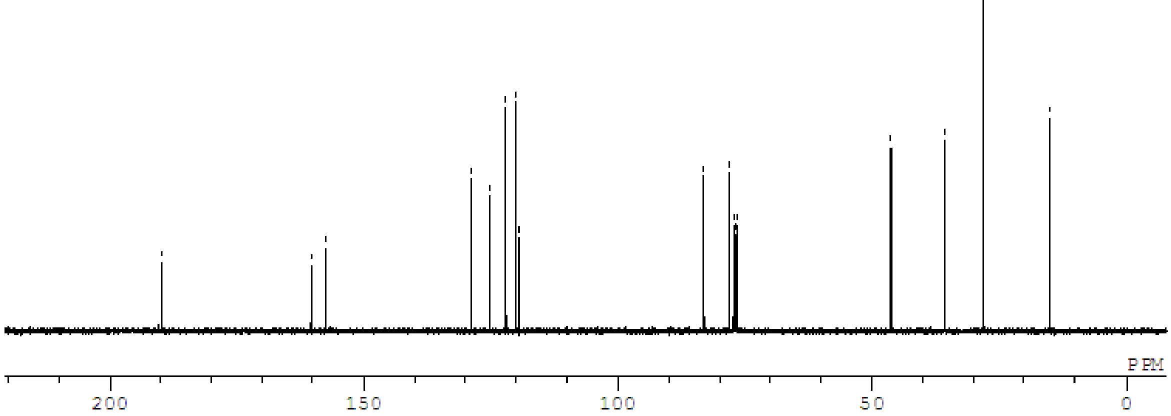

190.0
160.2
157.5
129.1
125.4
122.2
120.2
119.6
83.3
78.1
77.3
77.2
77.0
76.7
46.3
35.9
28.2
28.1
15.1
PPM
200
150
100
50
0

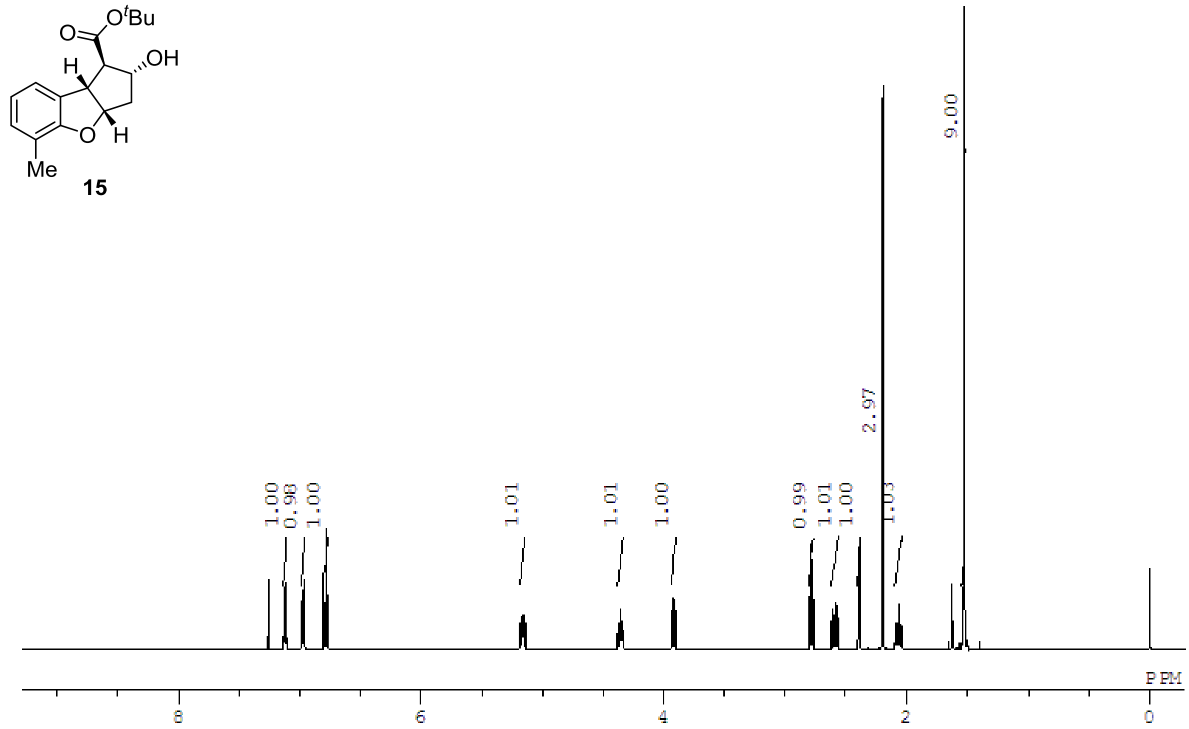
O^{t}Bu
OH
H
H
O
Me
15
1.00
0.96
1.00
1.01
1.01
1.00
0.99
1.01
1.00
1.03
2.97
9.00
PPM
8
6
4
2
0

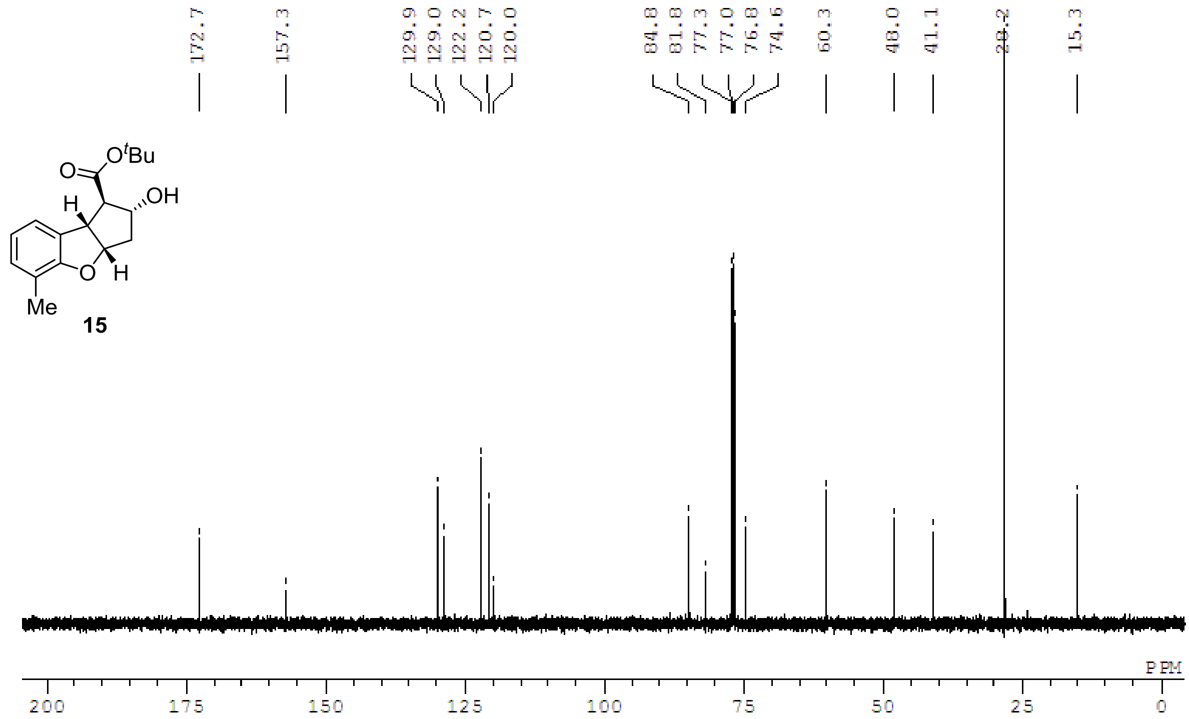
172.7
157.3
129.9
129.0
122.2
120.7
120.0
84.8
81.8
77.3
77.0
76.8
74.6
60.3
48.0
41.1
28.2
15.3
O^{t}Bu
OH
H
H
O
Me
15
PPM
200
175
150
125
100
75
50
25
0

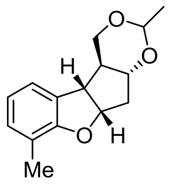

16

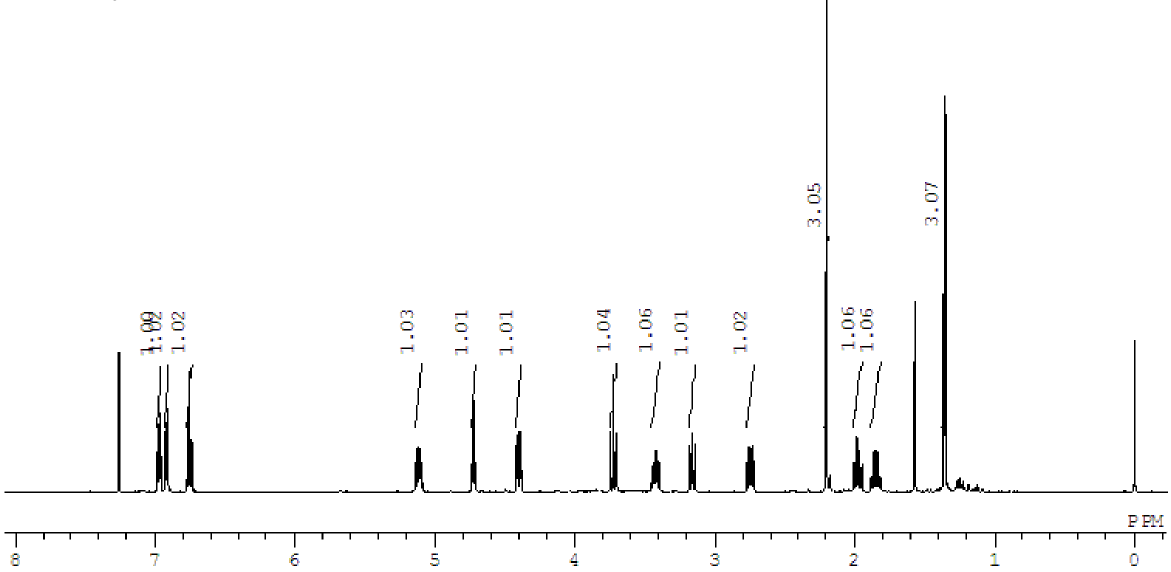

1.00
1.02
1.02
1.03
1.01
1.01
1.04
1.06
1.01
1.02
3.05
1.06
1.06
3.07
PPM
8
7
6
5
4
3
2
1
0

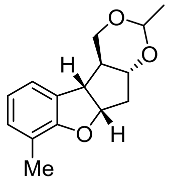

16

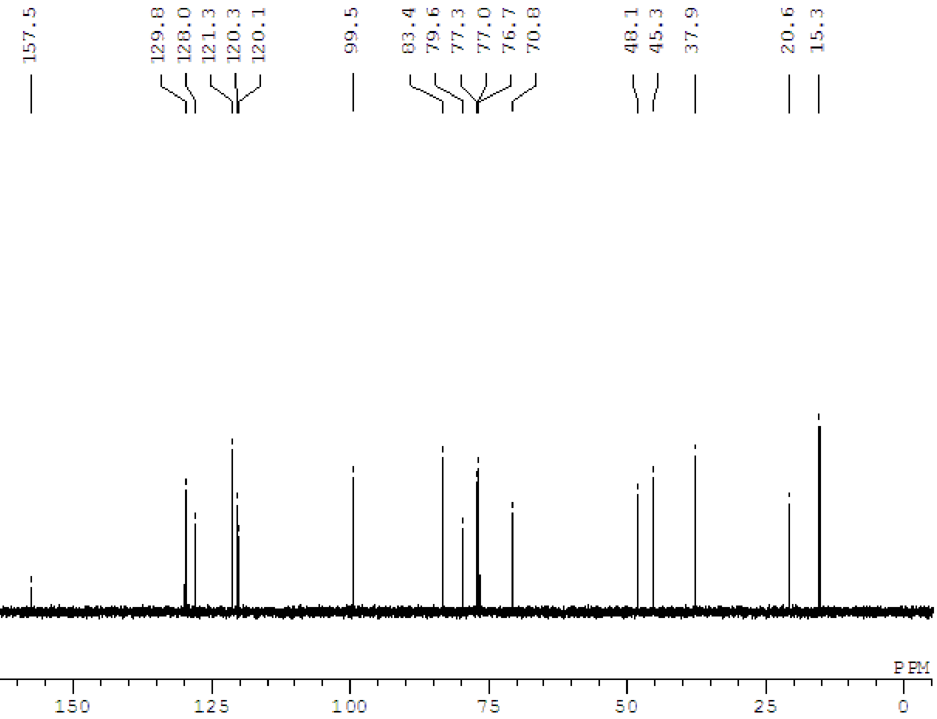

157.5
129.8
128.0
121.3
120.3
120.1
99.5
83.4
79.6
77.3
77.0
76.7
70.8
48.1
45.3
37.9
20.6
15.3
PPM
200
175
150
125
100
75
50
25
0

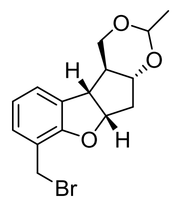
O
O
H
O
H
Br

17

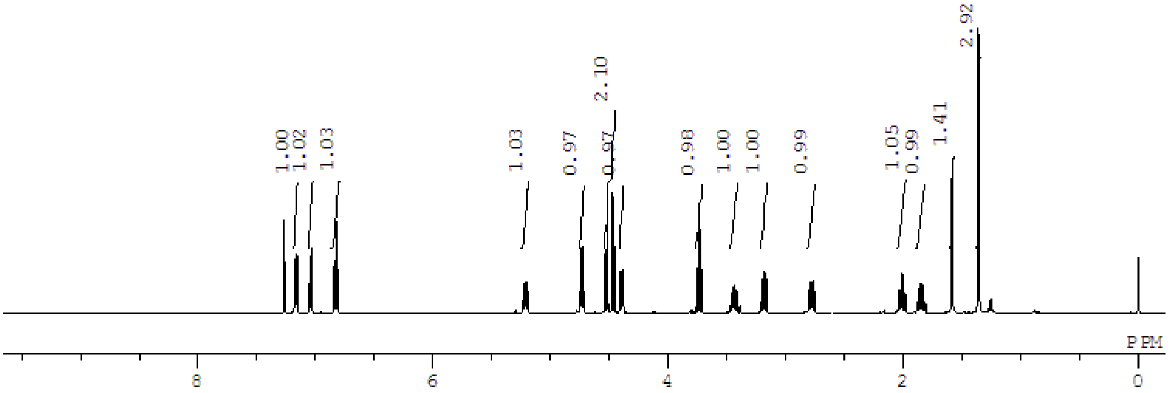
1.00
1.02
1.03
1.03
0.97
2.10
0.97
0.98
1.00
1.00
0.99
1.05
0.99
1.41
2.92
PPM
8
6
4
2
0

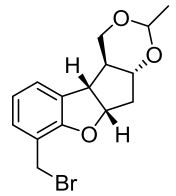
O
O
H
O
H
Br

17

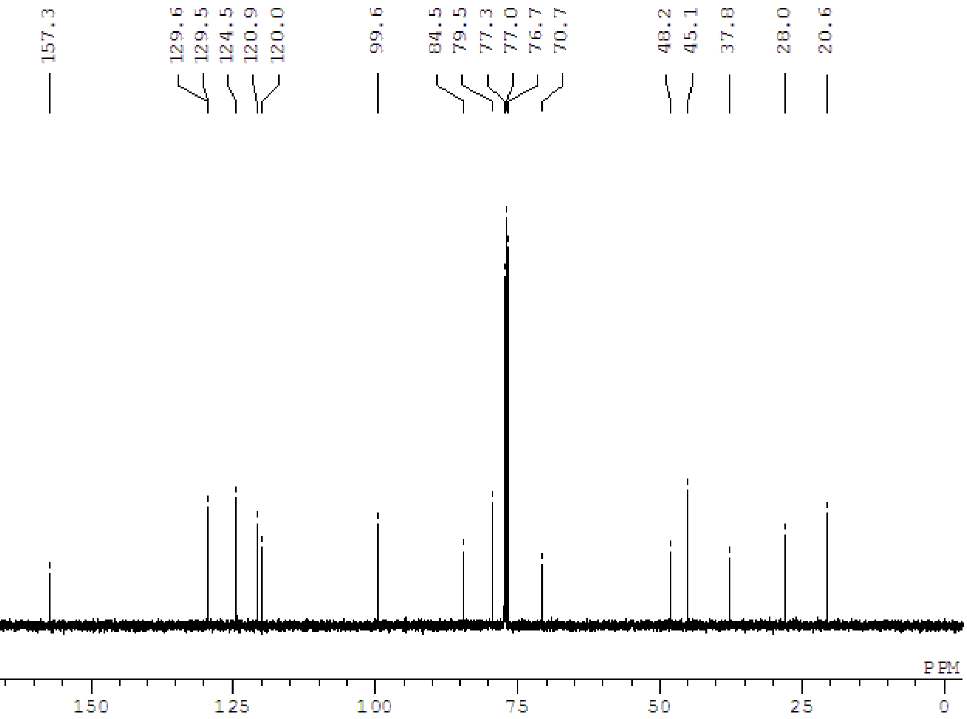
157.3
129.6
129.5
124.5
120.9
120.0
99.6
84.5
79.5
77.3
77.0
76.7
70.7
48.2
45.1
37.8
28.0
20.6
PPM
200
175
150
125
100
75
50
25
0

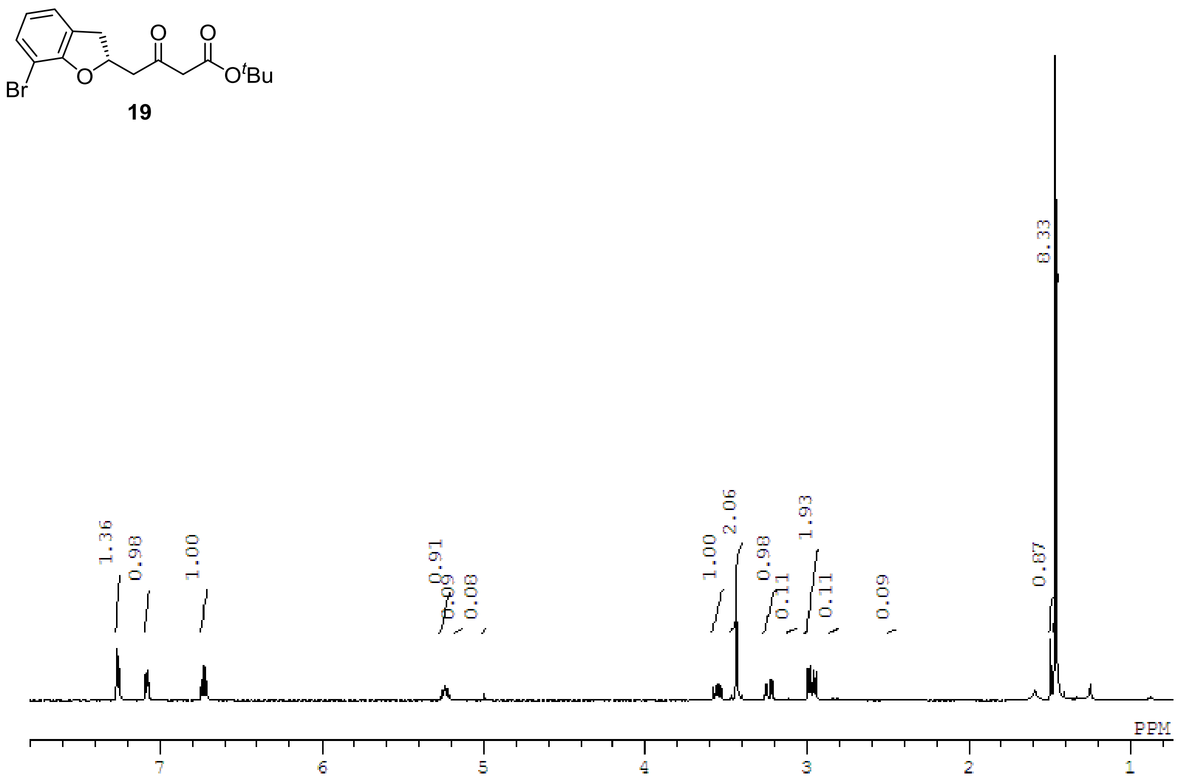
Br
O
O
O
O^{t}Bu
19
1.36
0.98
1.00
0.91
0.09
0.08
1.00
2.06
0.98
0.11
1.93
0.11
0.09
0.87
8.33
PPM
7
6
5
4
3
2
1

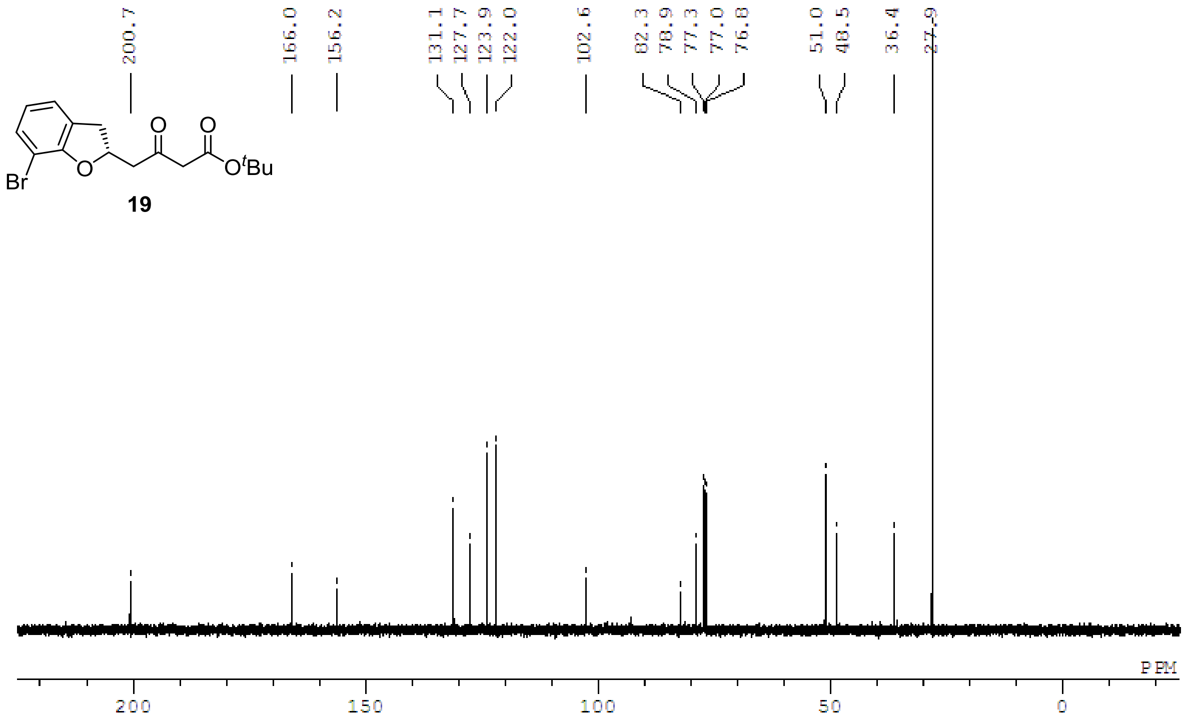
200.7
166.0
156.2
131.1
127.7
123.9
122.0
102.6
82.3
78.9
77.3
77.0
76.8
51.0
48.5
36.4
27.9
Br
O
O
O
O^{t}Bu
19
PPM
200
150
100
50
0

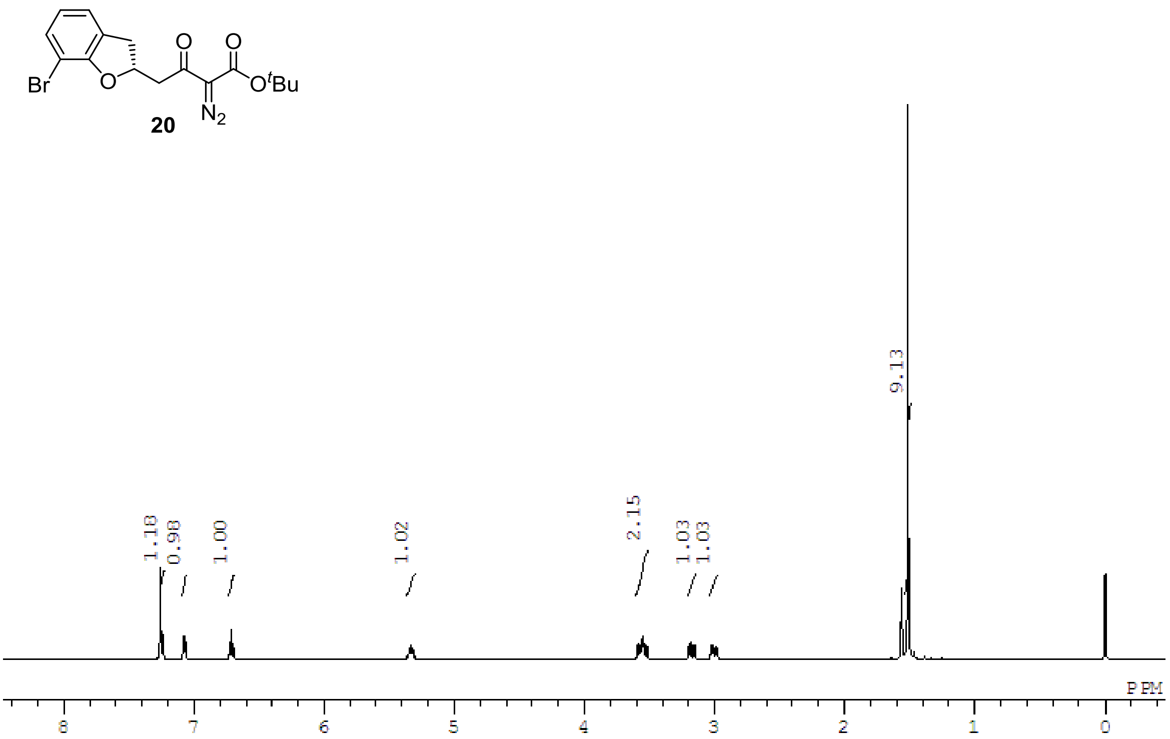

Br
O
O
O
O^tBu
N2
20
1.18
0.98
1.00
1.02
2.15
1.03
1.03
9.13
PPM
8
7
6
5
4
3
2
1
0

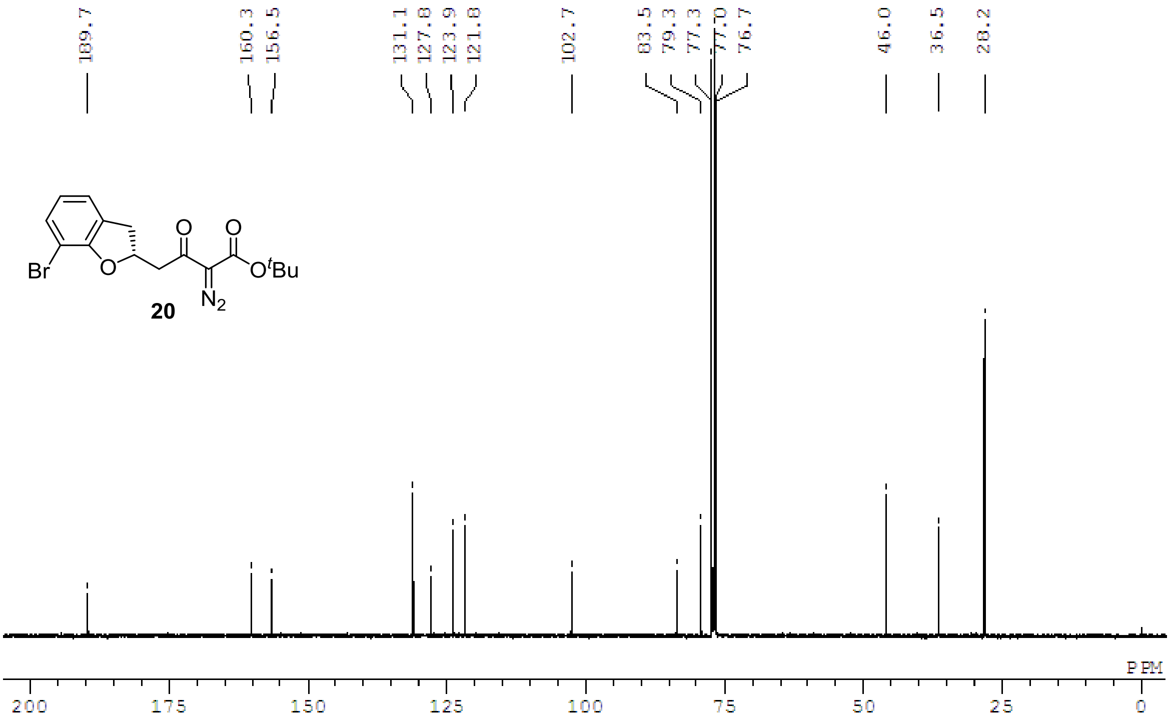

189.7
160.3
156.5
131.1
127.8
123.9
121.8
102.7
83.5
79.3
77.3
77.0
76.7
46.0
36.5
28.2
Br
O
O
O
O^tBu
N2
20
PPM
200
175
150
125
100
75
50
25
0

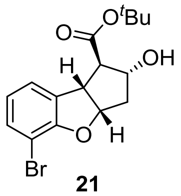
O
O^tBu
H
OH
O
H
Br

21

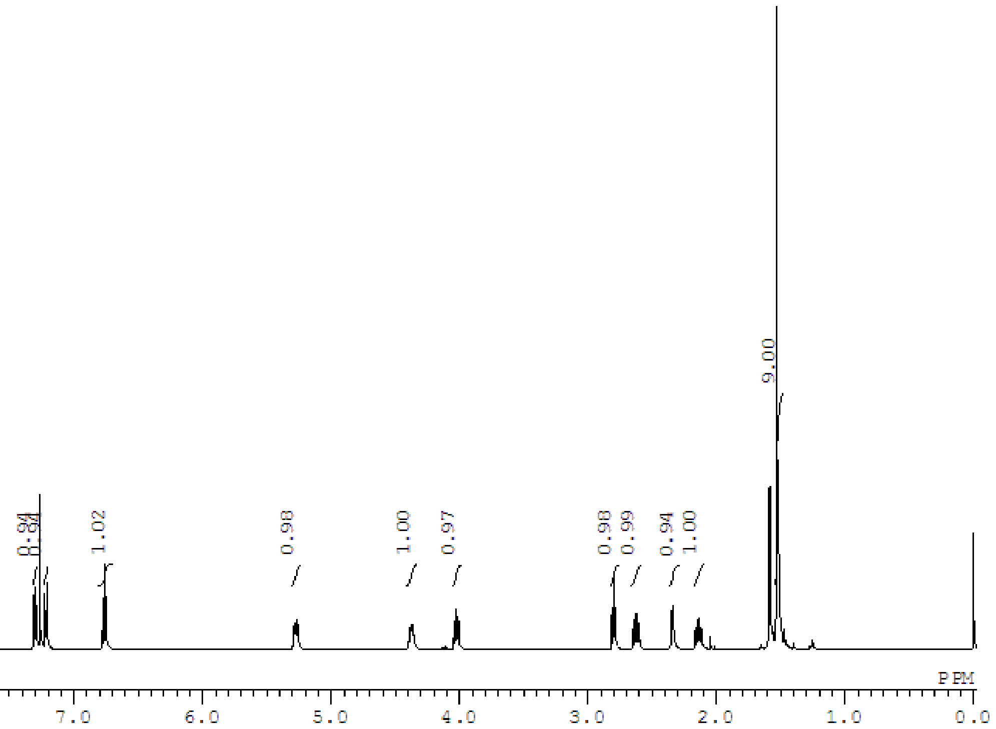
0.94
0.92
1.02
0.98
1.00
0.97
0.98
0.99
0.94
1.00
9.00
PPM
9.0 8.0 7.0 6.0 5.0 4.0 3.0 2.0 1.0 0.0

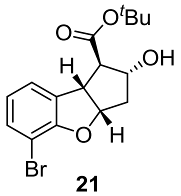
O
O^tBu
H
OH
O
H
Br

21

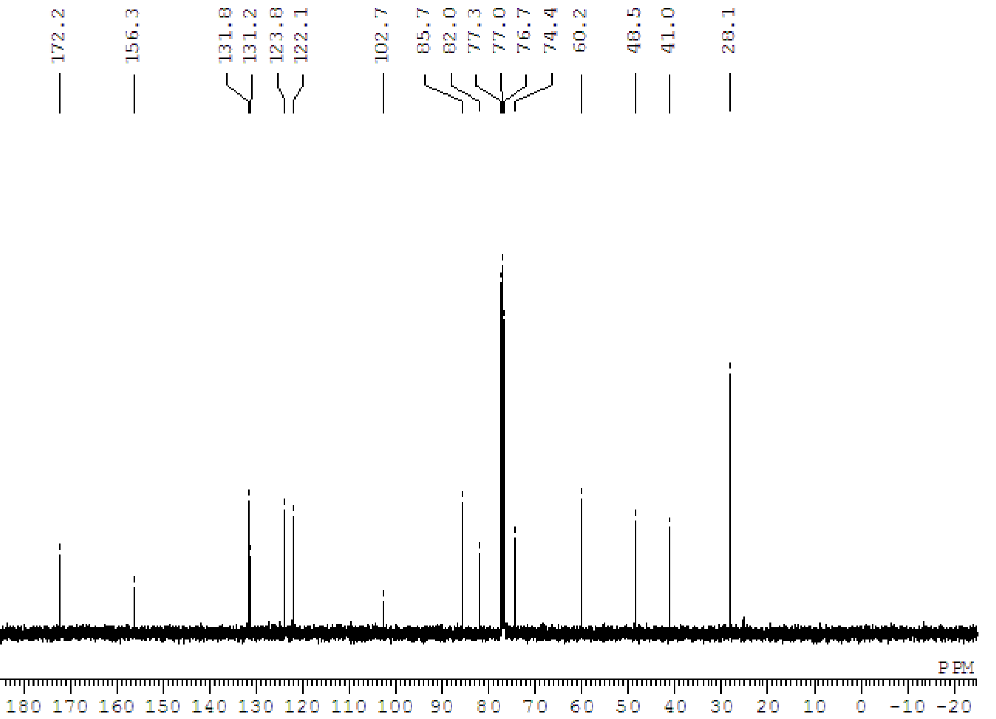
172.2
156.3
131.8
131.2
123.8
122.1
102.7
85.7
82.0
77.3
77.0
76.7
74.4
60.2
48.5
41.0
28.1
PPM
220 210 200 190 180 170 160 150 140 130 120 110 100 90 80 70 60 50 40 30 20 10 0 -10 -20

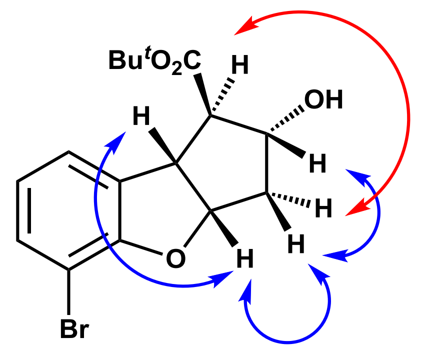

21

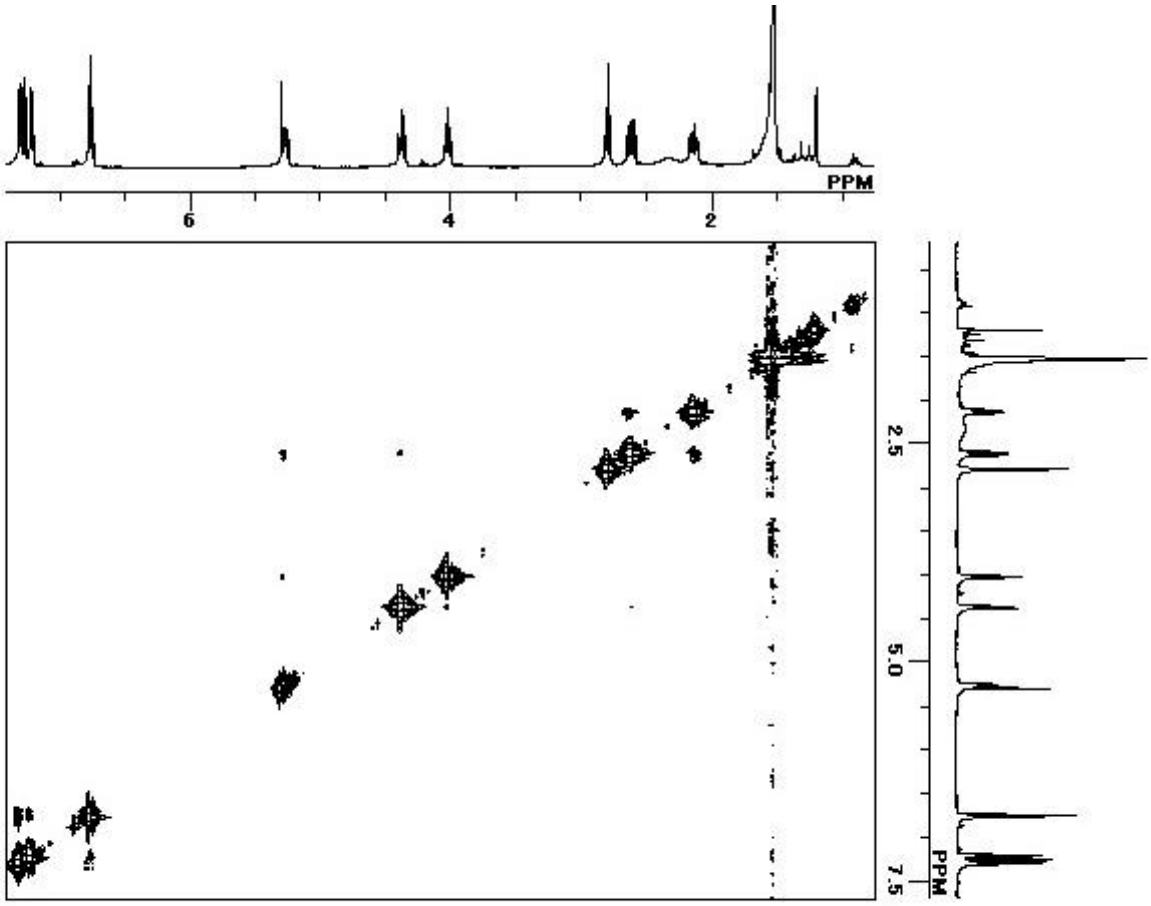

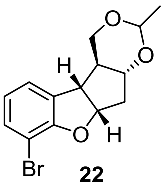

O
O
H
H
O
Br

22

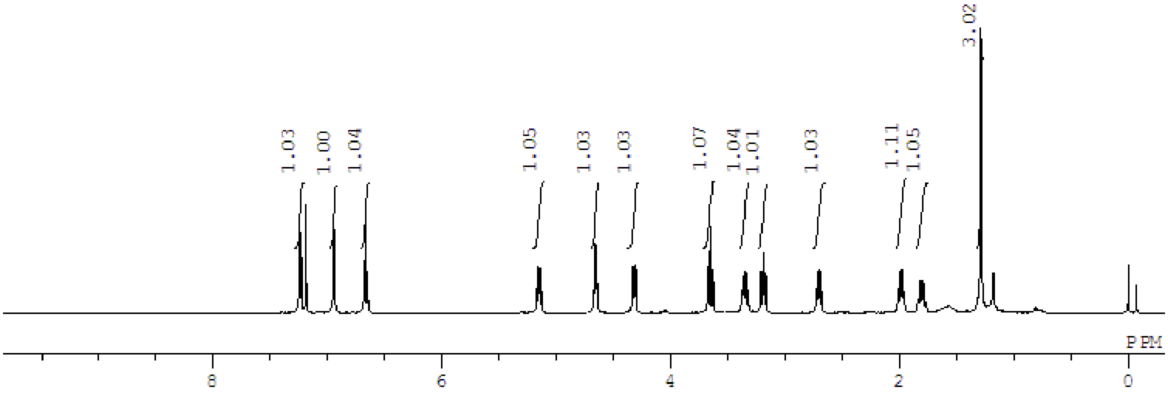

1.03
1.00
1.04
1.05
1.03
1.03
1.07
1.04
1.01
1.03
1.11
1.05
3.02
PPM
8
6
4
2
0

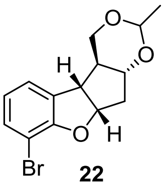

O
O
H
H
O
Br

22

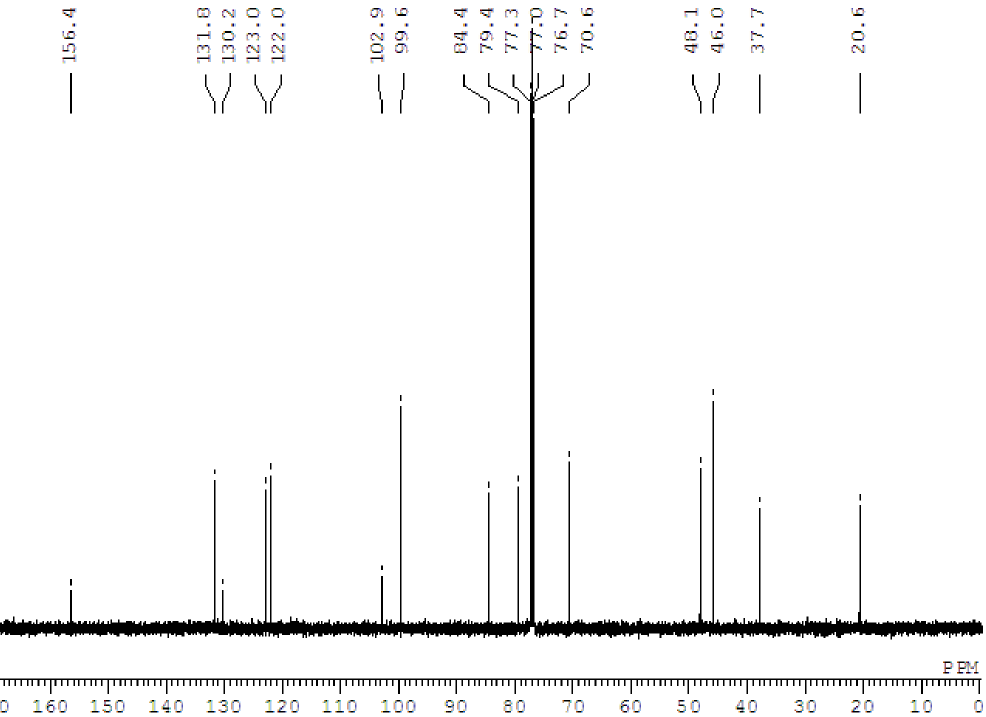

156.4
131.8
130.2
123.0
122.0
102.9
99.6
84.4
79.4
77.3
77.0
76.7
70.6
48.1
46.0
37.7
20.6
PPM
190
180
170
160
150
140
130
120
110
100
90
80
70
60
50
40
30
20
10
0

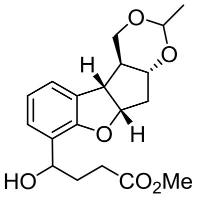
HO
CO_2Me
H
H
O
O
O

23

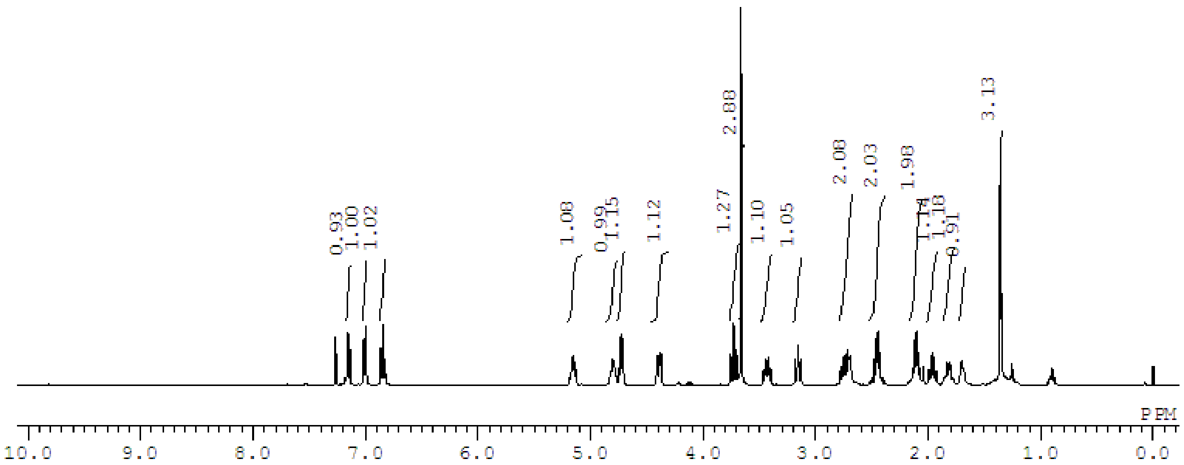
0.93
1.00
1.02
1.08
0.99
1.15
1.12
1.27
2.88
1.10
1.05
2.08
2.03
1.98
1.14
1.18
0.91
3.13
PPM
10.0
9.0
8.0
7.0
6.0
5.0
4.0
3.0
2.0
1.0
0.0

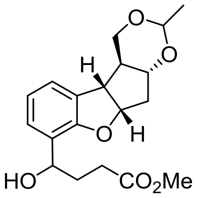
HO
CO_2Me
H
H
O
O
O

23

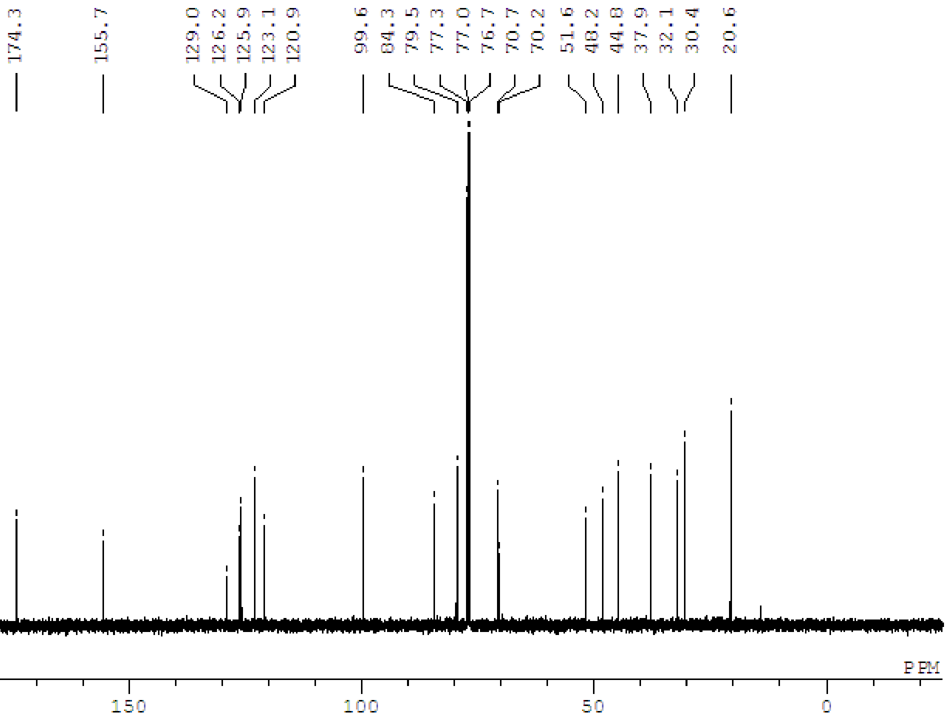
174.3
155.7
129.0
126.2
125.9
123.1
120.9
99.6
84.3
79.5
77.3
77.0
76.7
70.7
70.2
51.6
48.2
44.8
37.9
32.1
30.4
20.6
PPM
200
150
100
50
0

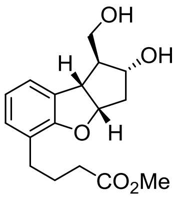
OH
OH
H
O
H
CO2Me

2

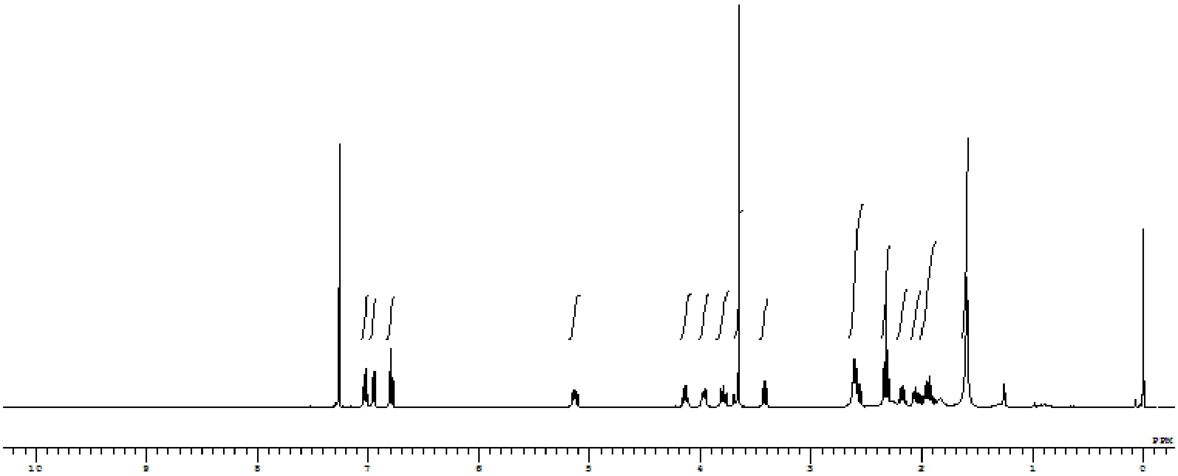
PPM
10
9
8
7
6
5
4
3
2
1
0

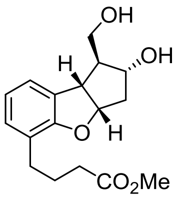
OH
OH
H
O
H
CO2Me

2

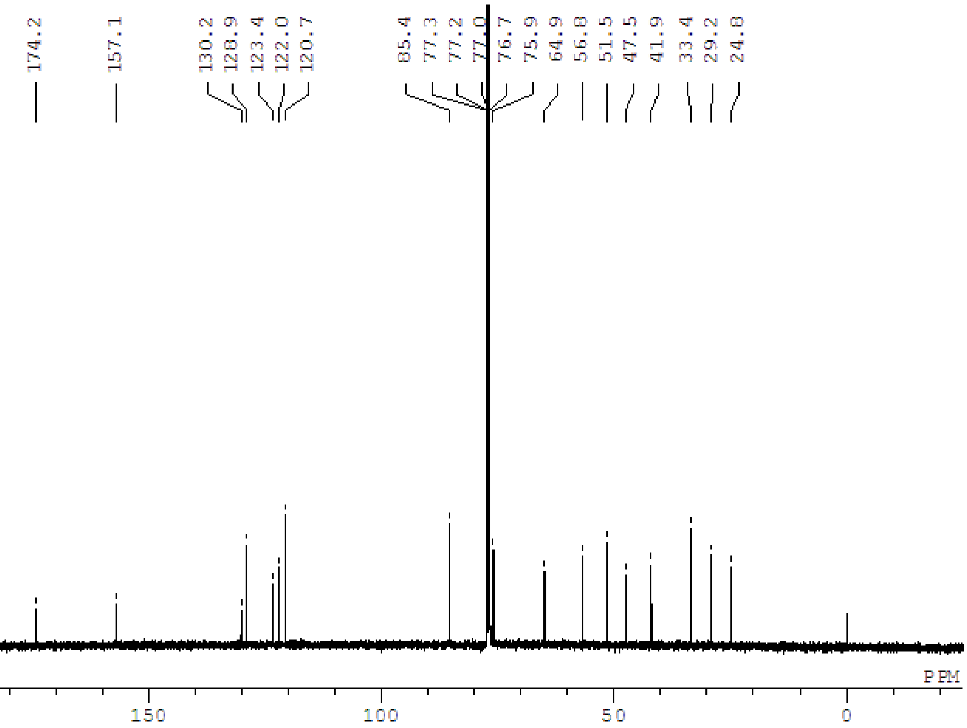
174.2
157.1
130.2
128.9
123.4
122.0
120.7
85.4
77.3
77.2
77.0
76.7
75.9
64.9
56.8
51.5
47.5
41.9
33.4
29.2
24.8
PPM
200
150
100
50
0

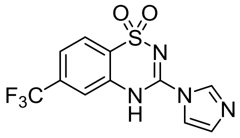
O O
S
N
F3C
N
H
N
N

S2

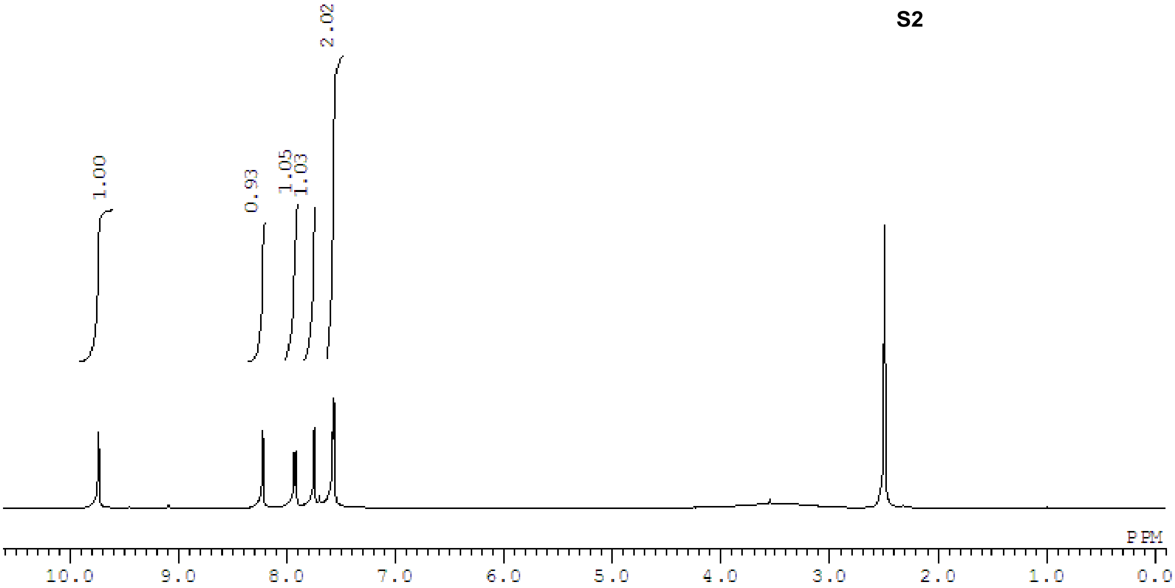
1.00
0.93
1.05
1.03
2.02
PPM
10.0 9.0 8.0 7.0 6.0 5.0 4.0 3.0 2.0 1.0 0.0

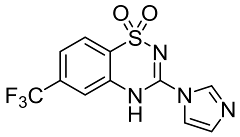
O O
S
N
F3C
N
H
N
N

S2

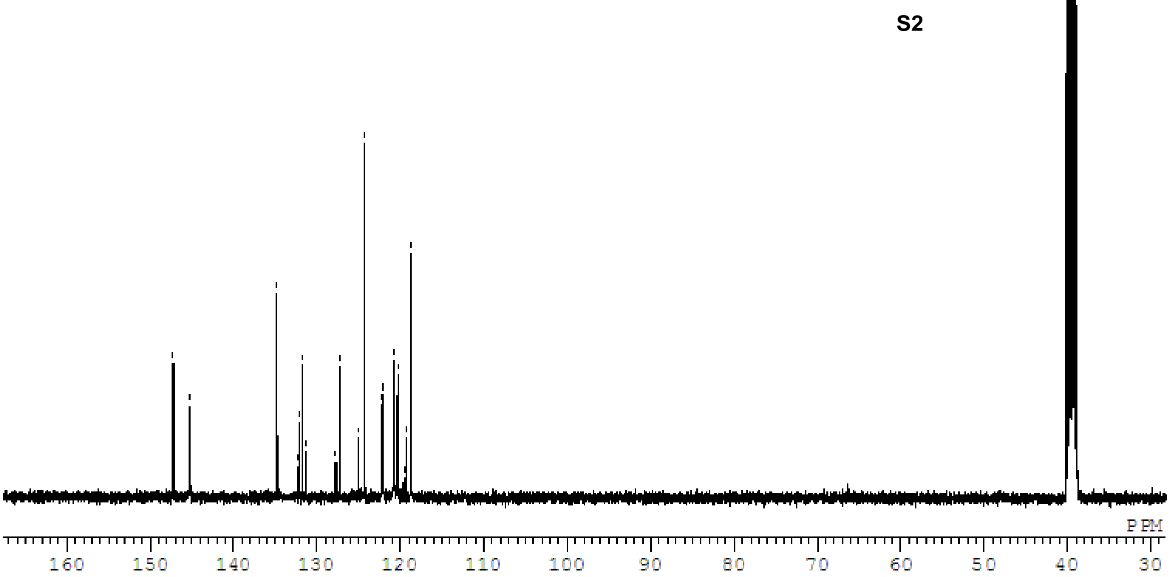
147.2
145.2
134.8
132.4
132.1
131.7
131.4
127.7
127.3
125.0
124.3
122.3
122.2
122.1
122.1
122.0
120.8
120.4
120.3
120.3
120.3
119.6
119.3
118.7
PPM
160 150 140 130 120 110 100 90 80 70 60 50 40 30

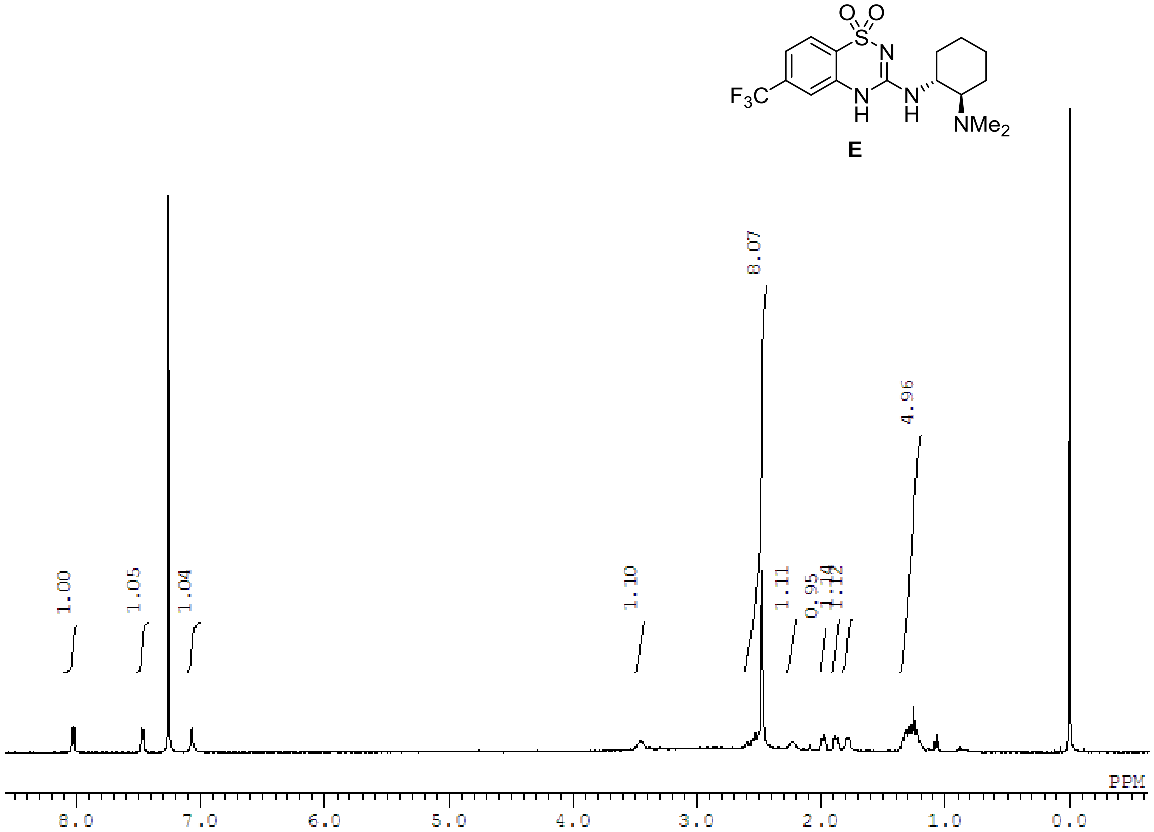

F3C
E
NMe2
1.00
1.05
1.04
1.10
8.07
1.11
0.95
4.96
PPM
8.0
7.0
6.0
5.0
4.0
3.0
2.0
1.0
0.0

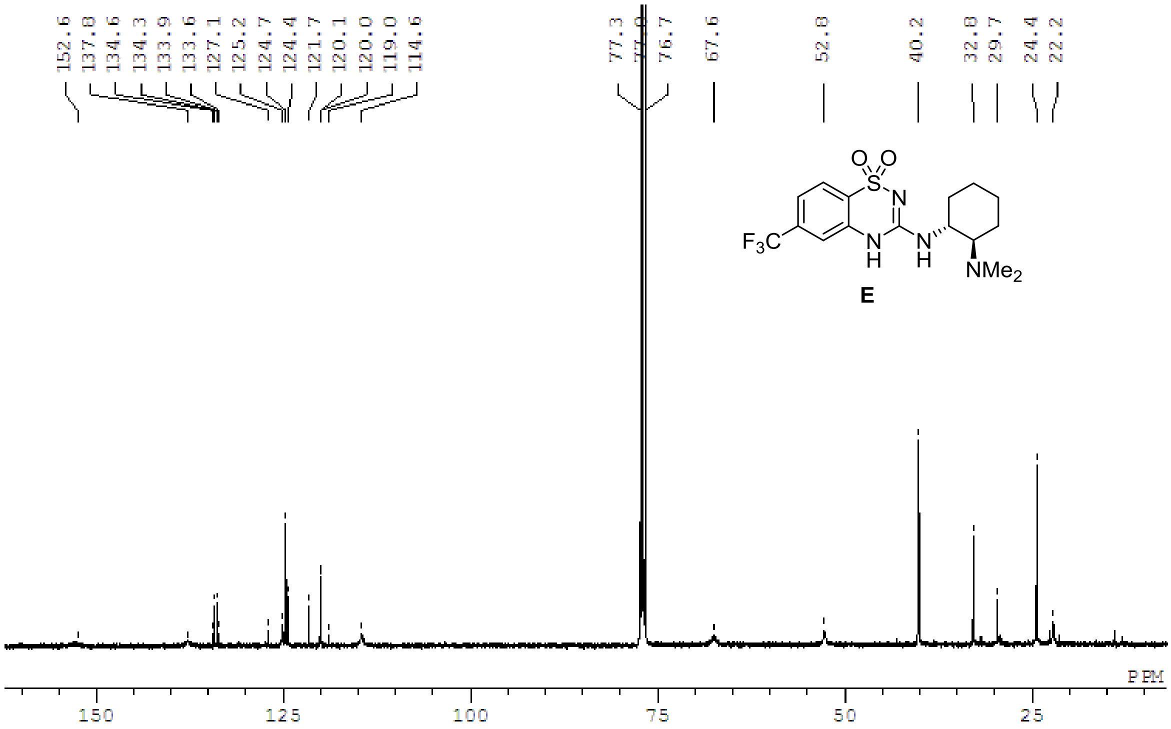

152.6
137.8
134.6
134.3
133.9
133.6
127.1
125.2
124.7
124.4
121.7
120.1
120.0
119.0
114.6
77.3
76.7
67.6
52.8
40.2
32.8
29.7
24.4
22.2
F3C
E
NMe2
PPM
150
125
100
75
50
25

5. Copies of HPLC charts

==== Shimadzu LCsolution Analysis Report ====

C:¥data_131217¥Kuramoto¥1-100¥10-racemi.lcd

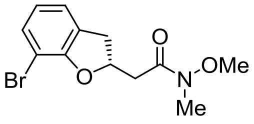

5

sample ID : 10
method name : 1.0-95-auto-shoki10-4-4.lcm
acquisition date : 2013/05/01 11:46:30
modified date : 2013/06/29 18:47:31

<Chromatogram>

chromatogram
10 C:¥data_131217¥Kuramoto¥1-100¥10-racemi.lcd

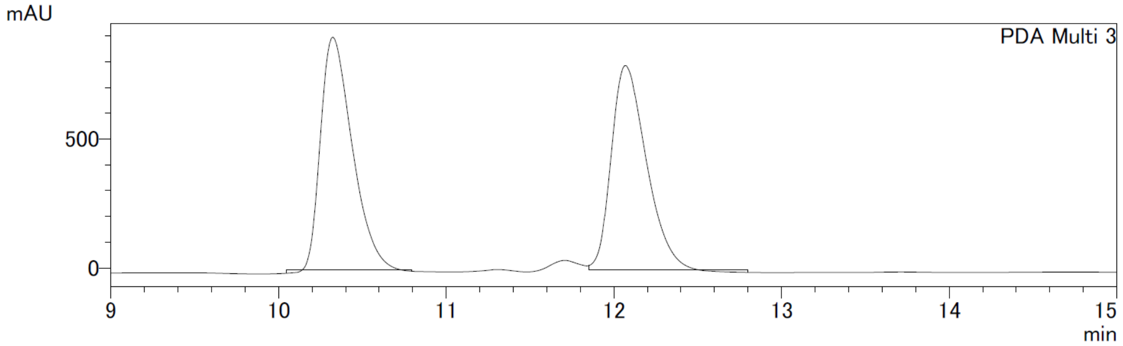

1 PDA Multi 3 / 220nm 4nm

<Peak Report>

peak table C:¥data_131217¥Kuramoto¥1-100¥10-racemi.lcd

PDA Ch3 220nm 4nm

peak#	retention time (min)	area	area (%)
1	10.318	11710592	50.834
2	12.064	11326264	49.166

==== Shimadzu LCsolution Analysis Report ====

C:¥data_131217¥Kuramoto¥101-200¥136(10)-IB-1.0-95.lcd

sample ID : 136(10)
method name : 95-IPA5-4.4-auto-shoki-30min.lcm
acquisition date : 2013/12/04 20:16:06
modified date : 2013/12/04 21:58:40

<Chromatogram>

chromatogram
136(10) C:¥data_131217¥Kuramoto¥101-200¥136(10)-IB-1.0-95.lcd

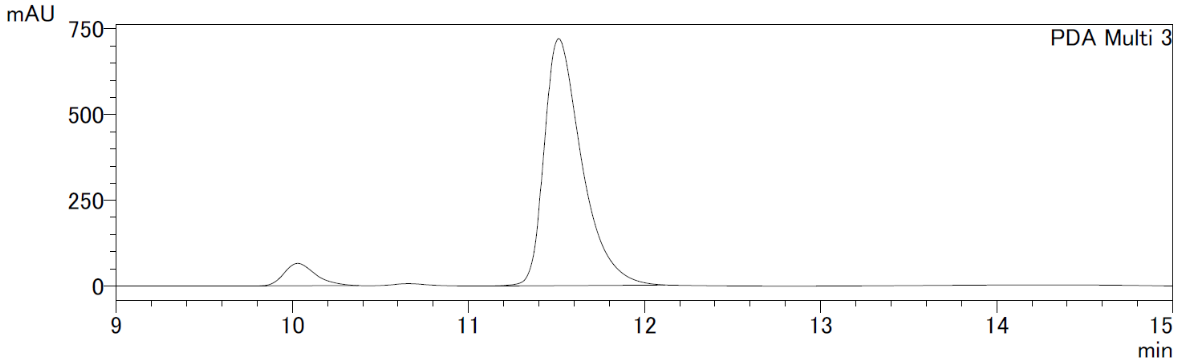

1 PDA Multi 3 / 220nm 4nm

<Peak Report>

peak table C:¥data_131217¥Kuramoto¥101-200¥136(10)-IB-1.0-95.lcd

PDA Ch3 220nm 4nm

peak#	retention time (min)	area	area (%)
1	10.025	806139	7.014
2	11.507	10687644	92.986

==== Shimadzu LCsolution Analysis Report ====

C:¥data_131217¥Kuramoto¥201-300¥275-IB-1.0-95.lcd

sample ID : rac-methyl
method name : 95-IPA5-4.4-auto-shoki-60min.lcm
acquisition date : 2014/05/27 16:57:48
modified date : 2014/05/28 11:52:55

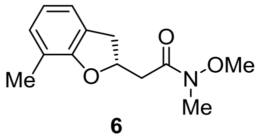

6

<Chromatogram>

chromatogram
rac-methyl C:¥data_131217¥Kuramoto¥201-300¥275-IB-1.0-95.lcd

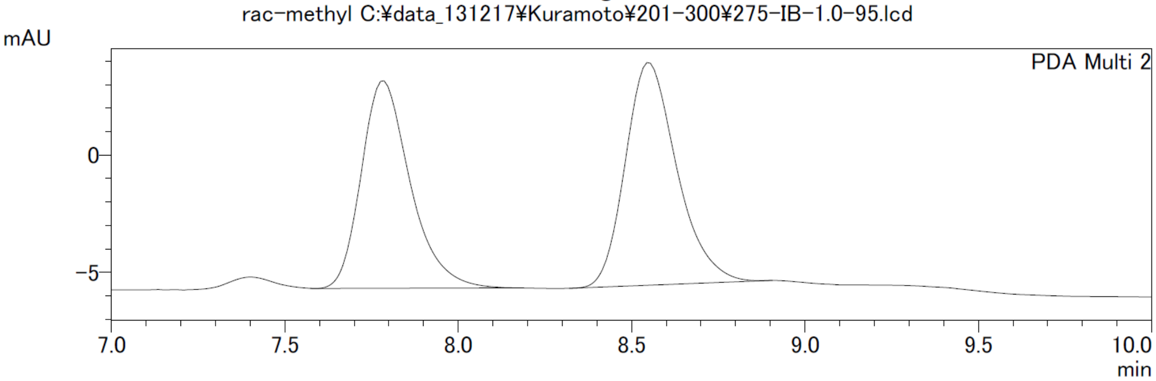

1 PDA Multi 2 / 238nm 4nm

<Peak Report>

peak table C:¥data_131217¥Kuramoto¥201-300¥275-IB-1.0-95.lcd

PDA Ch2 238nm 4nm

peak#	retention time (min)	area	area (%)
1	7.777	87048	46.948
2	8.543	98365	53.052

==== Shimadzu LCsolution Analysis Report ====

C:¥data_131217¥Kuramoto¥301-400¥315-IB-0.7-99.lcd

sample ID : 308(275)
method name : (275)95-IPA5-4.4-auto-shoki-20min.lcm
acquisition date : 2014/07/10 22:30:58
modified date : 2015/09/12 10:38:46

<Chromatogram>

chromatogram
308(275) C:¥data_131217¥Kuramoto¥301-400¥315-IB-0.7-99.lcd

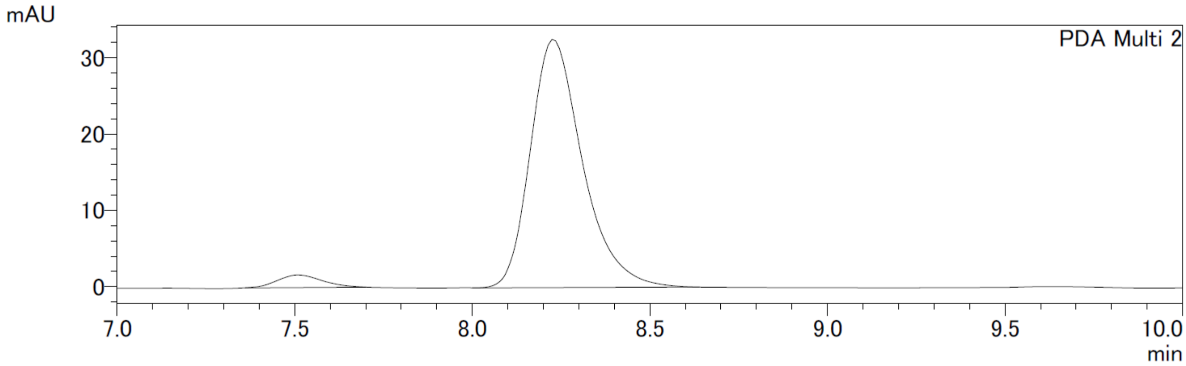

1 PDA Multi 2 / 238nm 4nm

<Peak Report>

peak table C:¥data_131217¥Kuramoto¥301-400¥315-IB-0.7-99.lcd

PDA Ch2 238nm 4nm

peak#	retention time (min)	area	area (%)
1	7.504	14565	4.204
2	8.222	331901	95.796

==== Shimadzu LCsolution Analysis Report ====

C:¥data_131217¥Kuramoto¥101-200¥115-IB-1.0.lcd

sample ID	: 112rac
method name	: 99-IPA.1.0-4.4-60min-1.0v-auto.lcm
acquisition date	: 2013/11/07 19:38:12
modified date	: 2013/11/09 10:37:48

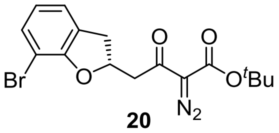

<Chromatogram>

chromatogram
112rac C:¥data_131217¥Kuramoto¥101-200¥115-IB-1.0.lcd

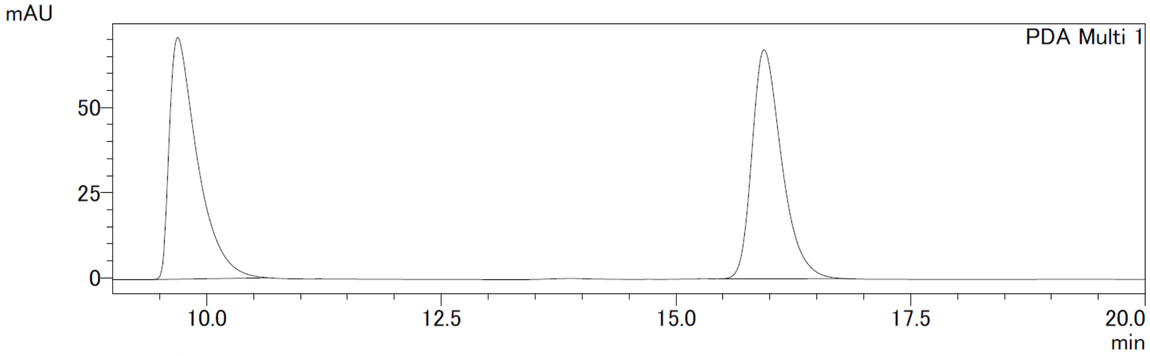

1 PDA Multi 1 / 254nm 4nm

<Peak Report>

peak table C:¥data_131217¥Kuramoto¥101-200¥115-IB-1.0.lcd

PDA Ch1 254nm 4nm

peak#	retention time (min)	area	area (%)
1	9.687	1475589	49.883
2	15.934	1482503	50.117

==== Shimadzu LCsolution Analysis Report ====

C:¥data_131217¥Kuramoto¥201-300¥266p(115)-IB-1.0-99.lcd

sample ID	: 266p(115)
method name	: (115)99-IPA.1.0-4.4-30min-1.0v-auto.lcm
acquisition date	: 2014/05/22 15:19:07
modified date	: 2014/05/26 16:43:50

<Chromatogram>

chromatogram
266p(115) C:¥data_131217¥Kuramoto¥201-300¥266p(115)-IB-1.0-99.lcd

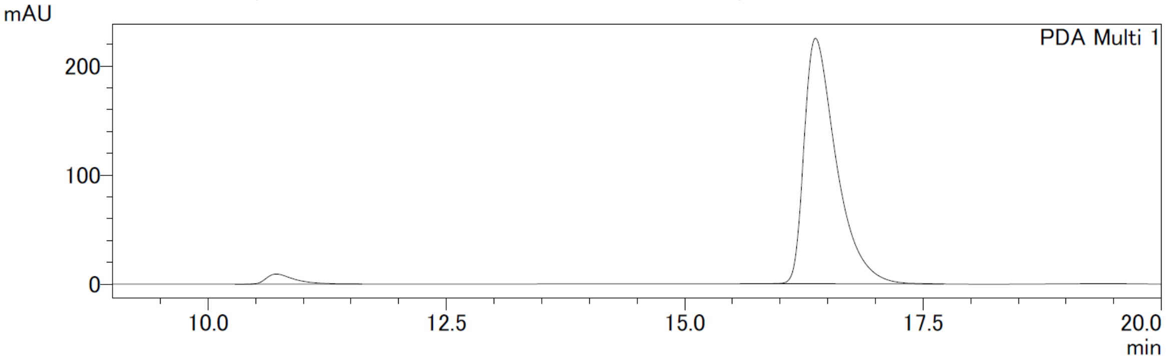

1 PDA Multi 1 / 254nm 4nm

<Peak Report>

peak table C:¥data_131217¥Kuramoto¥201-300¥266p(115)-IB-1.0-99.lcd

PDA Ch1 254nm 4nm

peak#	retention time (min)	area	area (%)
1	10.709	189238	3.462
2	16.366	5276934	96.538

==== Shimadzu LCsolution Analysis Report ====

C:¥data_131217¥Kuramoto¥201-300¥244-AD-1.0-96.lcd

sample ID : rac-formal
method name : 95-IPA5-2.2-auto-shoki-60min.lcm
acquisition date : 2014/04/25 16:28:57
modified date : 2015/09/11 22:12:50

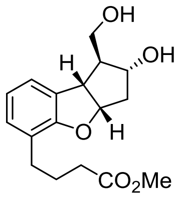

2

<Chromatogram>

chromatogram
rac-formal C:¥data_131217¥Kuramoto¥201-300¥244-AD-1.0-96.lcd

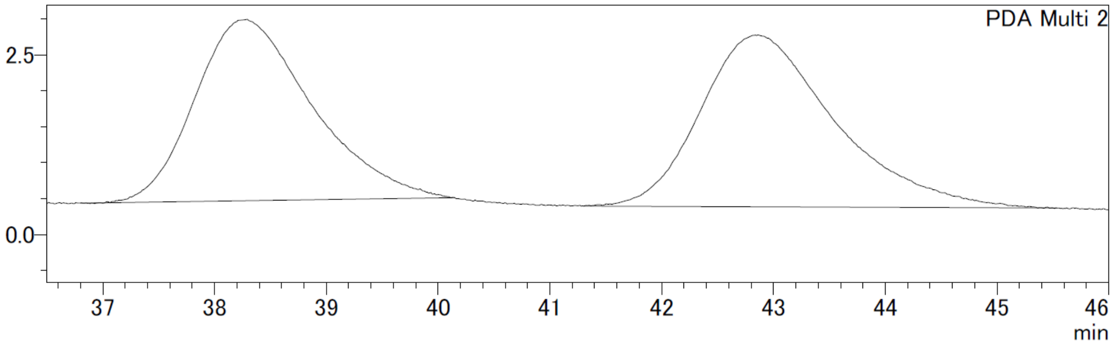

1 PDA Multi 2 / 238nm 4nm

<Peak Report>

peak table C:¥data_131217¥Kuramoto¥201-300¥244-AD-1.0-96.lcd

PDA Ch2 238nm 4nm

peak#	retention time (min)	area	area (%)
1	38.286	183781	47.956
2	42.831	199445	52.044

==== Shimadzu LCsolution Analysis Report ====

C:¥data_131217¥Kuramoto¥201-300¥297(244)-AD-1.0-95.lcd

sample ID : 297(244)
method name : (244)95-IPA5-2.2-auto-shoki-60min.lcm
acquisition date : 2014/07/10 18:24:18
modified date : 2015/09/11 21:32:56

<Chromatogram>

chromatogram
297(244) C:¥data_131217¥Kuramoto¥201-300¥297(244)-AD-1.0-95.lcd

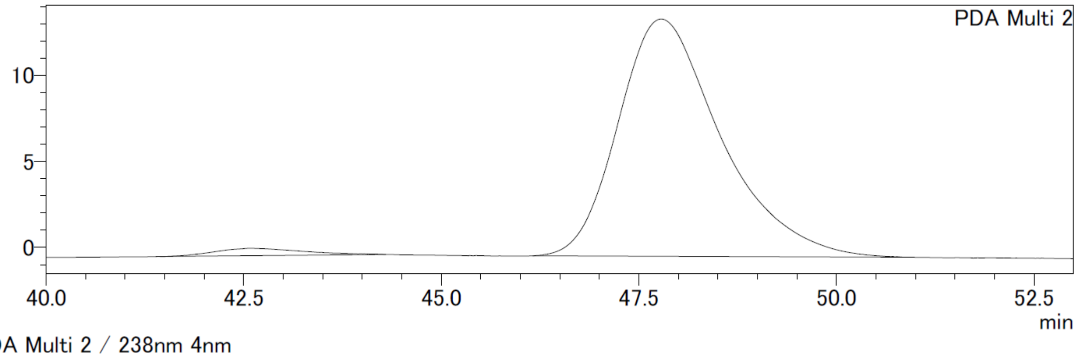

1 PDA Multi 2 / 238nm 4nm

<Peak Report>

peak table C:¥data_131217¥Kuramoto¥201-300¥297(244)-AD-1.0-95.lcd

PDA Ch2 238nm 4nm

peak#	retention time (min)	area	area (%)
1	42.596	32699	2.555
2	47.775	1246956	97.445

6. Computational details

The proposed transition states for oxa-Michael reactions (B3LYP/6-31G* level using Gaussian 09)

Benzothiadiazine-TS

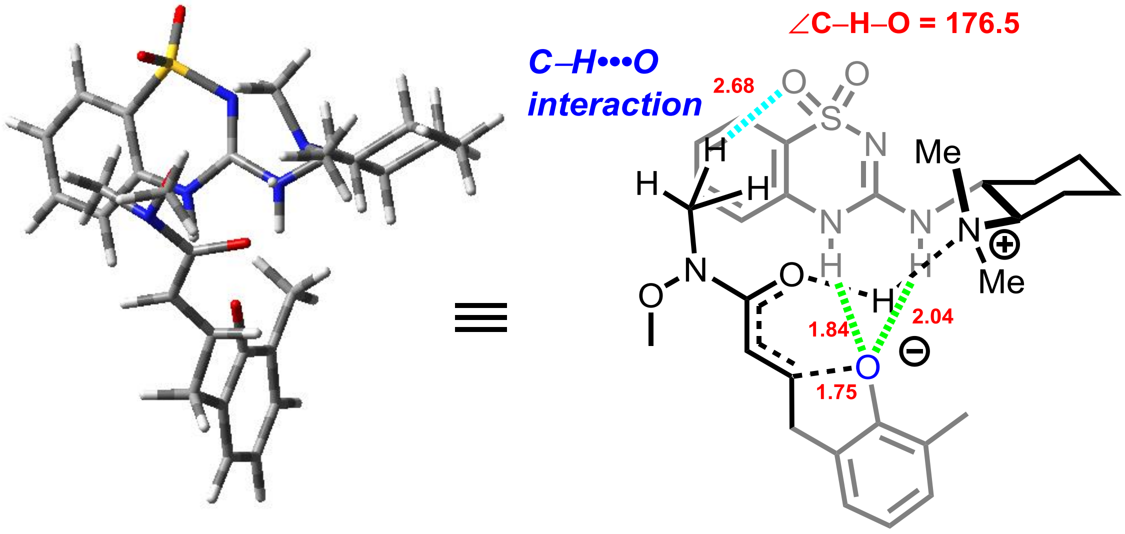

Thiourea-TS

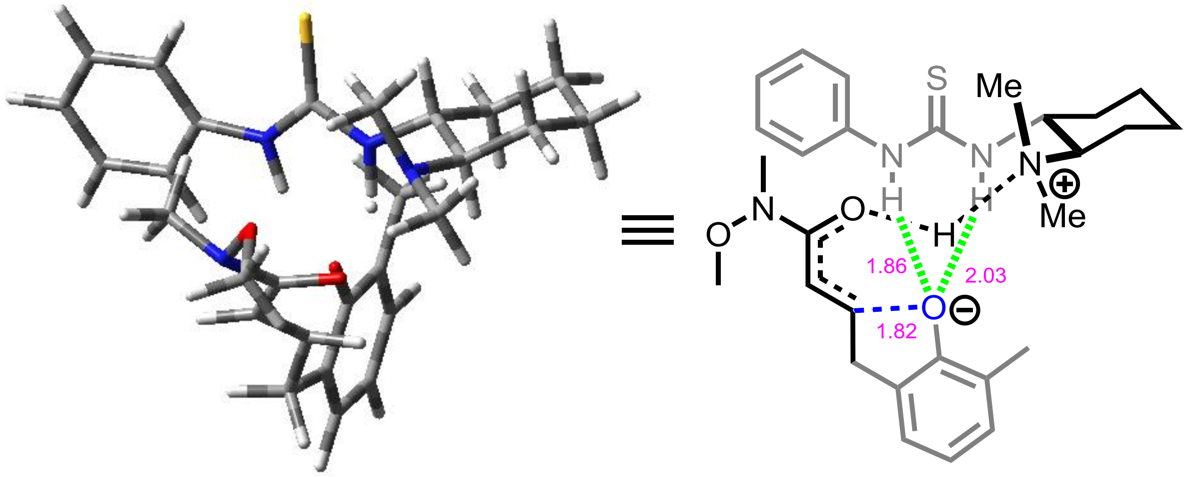

The energy profile of the reaction pathway estimated by DFT calculations at B3LYP/6-31G* and B3LYP/6-311+G**// B3LYP/6-31G* level using Gaussian 09)

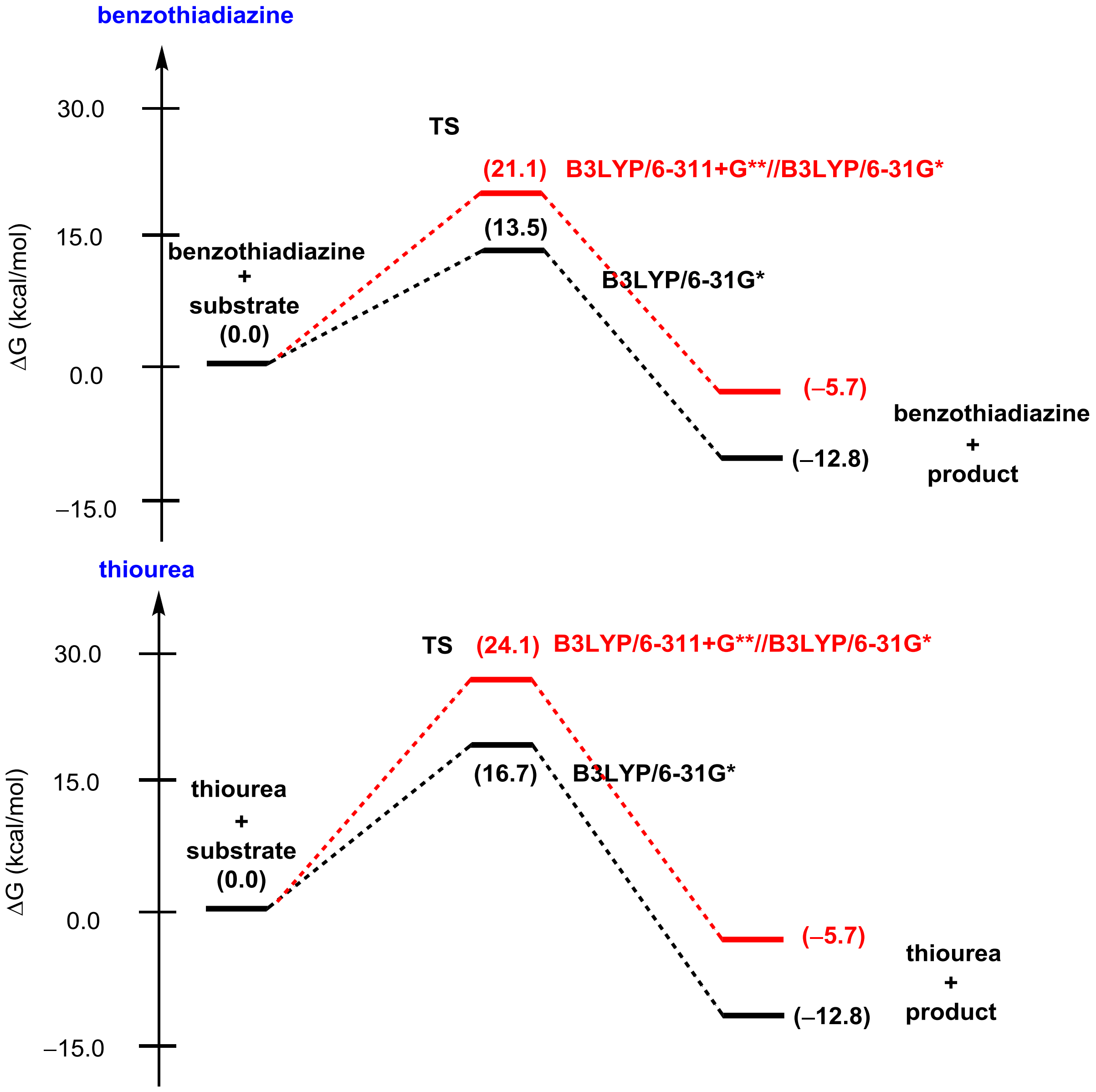

Cartesian coordinates and total energies for all the calculated structures

Substrate 8

Zero-point vibrational energy 740289.1 (Joules/Mol)

176.93334 (Kcal/Mol)

Zero-point correction= 0.281961 (Hartree/Particle)

Thermal correction to Energy= 0.300108

Thermal correction to Enthalpy= 0.301053

Thermal correction to Gibbs Free Energy= 0.233570

Sum of electronic and zero-point Energies= -785.685771

Sum of electronic and thermal Energies= -785.667624

Sum of electronic and thermal Enthalpies= -785.666680

Sum of electronic and thermal Free Energies= -785.734162

E(RB3LYP) = -785.967732157

E(RB3LYP/6-311+G**) = -786.196320217

0 1

C	0.30861700	-1.20931800	0.54473000
C	1.34822000	-0.39537500	0.76338400
C	2.54184200	-0.45484200	-0.12672100
O	2.72128300	-1.35623200	-0.93492500
C	-0.94736500	-1.25064500	1.37578100
C	-2.17699300	-0.86567700	0.57089100
C	-4.41157600	-0.11924300	-0.95085400
C	-2.47917300	0.48774100	0.34774900
C	-3.01945300	-1.83082100	0.01334900
C	-4.13232200	-1.46909300	-0.74526600
C	-3.59706200	0.88024800	-0.40910300
O	-1.63484400	1.41063800	0.91542300
N	3.51742200	0.52404300	0.10002900
C	3.23669100	1.91160800	0.44263700
O	4.56105200	0.51514500	-0.85053800
C	5.57976400	-0.40862800	-0.46489200
C	-3.89643600	2.34616800	-0.62411300
H	-3.08016400	2.86557500	-1.14733500
H	-4.07399700	2.87855100	0.32204000
H	-4.79564300	2.47315400	-1.23315800

H	4.11501300	2.34088100	0.93236500
H	2.38891600	1.96856700	1.12487200
H	3.01090200	2.49874900	-0.45733300
H	5.96963900	-0.17408400	0.53381000
H	6.36963800	-0.28088200	-1.21002500
H	5.20511700	-1.43564800	-0.49466000
H	1.32681600	0.29986500	1.59690000
H	-0.84168700	-0.59076900	2.24310800
H	-1.08698700	-2.27255500	1.75419300
H	-2.79648600	-2.88194800	0.18236800
H	-5.27789600	0.17315700	-1.53973200
H	-4.77727700	-2.23239500	-1.17048200
H	0.36398600	-1.88903100	-0.30436000
H	-1.95029400	2.30135700	0.70211200

Product 6

Zero-point vibrational energy 746086.3 (Joules/Mol)

178.31891 (Kcal/Mol)

Zero-point correction= 0.284169 (Hartree/Particle)

Thermal correction to Energy= 0.301237

Thermal correction to Enthalpy= 0.302181

Thermal correction to Gibbs Free Energy= 0.237638

Sum of electronic and zero-point Energies= -785.708093

Sum of electronic and thermal Energies= -785.691026

Sum of electronic and thermal Enthalpies= -785.690081

Sum of electronic and thermal Free Energies= -785.754624

E(RB3LYP) = -785.992262589

E(RB3LYP/6-311+G**) = -786.209488474

0 1

C	0.12912900	0.16896500	0.64672500
C	-1.09240500	0.81013000	-0.00151000
C	-2.37169800	0.05882200	0.36467100
O	-2.37474200	-0.87244000	1.15715100
C	0.53213800	-1.22472100	0.10042700
C	2.02687400	-1.07603100	-0.06564600

C	4.63442700	-0.16843300	-0.33029700
C	2.34794800	0.27012000	0.10534800
C	3.02794700	-1.98886000	-0.37767600
C	4.34226100	-1.52434200	-0.50628200
C	3.64398900	0.77445300	-0.01754400
O	1.26233900	1.05371400	0.40172400
N	-3.51374700	0.44134100	-0.31901800
C	-3.74609100	1.74383700	-0.92240800
O	-4.71512500	-0.06132300	0.21559000
C	-5.02883400	-1.33123500	-0.36350800
C	3.93446600	2.23973800	0.17973000
H	3.41257600	2.85603400	-0.56325500
H	5.00664800	2.44237000	0.09539900
H	3.59665600	2.58385500	1.16485400
H	-4.50186800	1.63744400	-1.70493400
H	-2.82517000	2.11139600	-1.37677100
H	-4.10359600	2.47249700	-0.18277500
H	-5.10138800	-1.26176300	-1.45631700
H	-6.00316600	-1.59579900	0.05532400
H	-4.28557300	-2.08089100	-0.07842200
H	-1.16523700	1.85366600	0.33203800
H	0.04104200	-1.43517000	-0.86084200
H	0.23221200	-2.01652800	0.79152600
H	2.79767500	-3.04219400	-0.51703300
H	5.66202300	0.17402800	-0.43197600
H	5.14227600	-2.22022700	-0.74269200
H	-0.01399500	0.11934200	1.73100700
H	-0.95556400	0.83827600	-1.08990400

Benzothiadiazine catalyst C

Zero-point vibrational energy 976455.3 (Joules/Mol)

233.37843 (Kcal/Mol)

Zero-point correction= 0.371912 (Hartree/Particle)

Thermal correction to Energy= 0.392165

Thermal correction to Enthalpy= 0.393109

Thermal correction to Gibbs Free Energy= 0.322992

Sum of electronic and zero-point Energies= -1352.060099
Sum of electronic and thermal Energies= -1352.039846
Sum of electronic and thermal Enthalpies= -1352.038902
Sum of electronic and thermal Free Energies= -1352.109019
E(RB3LYP) = -1352.43201124
E(RB3LYP/6-311+G**) = -1352.71927733

0 1

H	-1.18071200	1.46050600	-0.54854300
N	-0.95035900	0.46707800	-0.61002600
C	3.17907900	-0.08418300	0.13971300
C	-2.06264000	-0.34047100	-0.09871100
C	-4.54497800	-0.24861100	0.36642600
C	-3.45873500	-2.43391900	-0.34029500
C	-4.73831400	-1.58543100	-0.37002800
C	-2.24899700	-1.65389600	-0.87611500
C	-3.33940600	0.52550800	-0.20466700
H	-4.38676900	-0.44302000	1.43581700
H	-3.25565600	-2.75230200	0.69222200
H	-5.01643600	-1.38391100	-1.41492000
H	-2.39376800	-1.41510400	-1.93911000
H	-3.52067100	0.67808200	-1.27877400
H	-1.87085800	-0.59488600	0.95350800
H	-5.45293900	0.36221500	0.28653800
H	-3.59715100	-3.35118700	-0.92487100
H	-5.57585200	-2.13782000	0.07368000
H	-1.33747200	-2.25227400	-0.79712000
N	-3.10162500	1.88135400	0.33402300
C	-4.04227800	2.87672000	-0.17128300
H	-3.71051200	3.87540300	0.13448300
H	-5.07447200	2.74055900	0.19804200
H	-4.06455600	2.84570800	-1.26575400
C	-2.98222700	1.96018300	1.79003700
H	-2.25526000	1.22890200	2.15387100
H	-3.93573000	1.79243400	2.31954600
H	-2.62006800	2.95728700	2.06387000
N	0.63460800	-0.94213000	0.33469200

C	0.34841100	0.12550900	-0.36004600
N	1.27692500	1.00558500	-0.88499500
C	2.62629000	1.02011600	-0.52252900
H	0.93280800	1.75589500	-1.46832300
C	4.50386500	-0.06266700	0.57597900
C	5.29919000	1.05093800	0.32006200
H	6.33329400	1.06948200	0.64979700
C	4.75889600	2.14299800	-0.36866800
H	5.37493900	3.01371800	-0.57474200
C	3.42953400	2.13961000	-0.77951800
H	3.00667500	3.00267500	-1.28855400
H	4.89363500	-0.92427000	1.10869700
S	2.17869600	-1.55323000	0.30893800
O	2.35049100	-2.36343700	-0.90424200
O	2.45768700	-2.15581800	1.61294700

Thiourea catalyst A

Zero-point vibrational energy 966825.3 (Joules/Mol)

231.07679 (Kcal/Mol)

Zero-point correction= 0.368244 (Hartree/Particle)

Thermal correction to Energy= 0.386912

Thermal correction to Enthalpy= 0.387856

Thermal correction to Gibbs Free Energy= 0.320181

Sum of electronic and zero-point Energies= -1147.532254

Sum of electronic and thermal Energies= -1147.513586

Sum of electronic and thermal Enthalpies= -1147.512642

Sum of electronic and thermal Free Energies= -1147.580317

E(RB3LYP) = -1147.90049817

E(RB3LYP/6-311+G**) = -1148.12987606

0 1

H	-0.54806900	1.03311100	-0.58308800
N	-0.49657600	0.01783900	-0.48146800
C	3.71292000	-0.18065200	0.92610700
C	-1.79508700	-0.51514400	-0.07353800
C	-4.22086900	0.11458000	0.22583200

C	-3.58843100	-2.26830400	-0.39517300
C	-4.65618500	-1.16908300	-0.50229200
C	-2.21036500	-1.77193900	-0.85805700
C	-2.84929600	0.59820100	-0.28791800
H	-4.16468500	-0.07977200	1.30546900
H	-3.51659000	-2.60559800	0.64879700
H	-4.83289800	-0.93724700	-1.56300900
H	-2.23397900	-1.52824200	-1.92980400
H	-2.93781700	0.74058400	-1.37556900
H	-1.74494100	-0.78666300	0.99041200
H	-4.97247300	0.90130900	0.08437200
H	-3.88640000	-3.14490300	-0.98331600
H	-5.61300800	-1.52305600	-0.09843700
H	-1.45314200	-2.54775700	-0.71575100
N	-2.36525900	1.89650700	0.22091700
C	-3.07921200	3.03969000	-0.33693600
H	-2.55563400	3.96228500	-0.06173000
H	-4.12241800	3.13055500	0.01588200
H	-3.09458500	2.97028100	-1.42998000
C	-2.25703300	1.99427400	1.67569800
H	-1.67021500	1.16060600	2.07092800
H	-3.23356900	2.00614200	2.19031700
H	-1.73409000	2.92236200	1.93128800
C	0.70547500	-0.47304100	-0.04254700
N	1.74826200	0.30009300	-0.51137300
C	3.14212700	0.22594300	-0.28783600
H	1.49788000	0.89792800	-1.29046900
C	5.09948800	-0.16559700	1.07297900
C	5.93155300	0.26253600	0.03725000
H	7.01013300	0.27163100	0.16596100
C	5.36052600	0.68140400	-1.16523400
H	5.98981700	1.02000900	-1.98393400
C	3.97766200	0.65985000	-1.32864400
H	3.53816200	0.97508900	-2.27324900
H	5.53026400	-0.48755800	2.01738400
S	0.86414900	-1.84618900	0.91102800
H	3.07725900	-0.50944200	1.73639900

TS (catalyst C)

Zero-point vibrational energy 1725582.1 (Joules/Mol)

412.42401 (Kcal/Mol)

Zero-point correction= 0.657240 (Hartree/Particle)

Thermal correction to Energy= 0.695225

Thermal correction to Enthalpy= 0.696169

Thermal correction to Gibbs Free Energy= 0.586205

Sum of electronic and zero-point Energies= -2137.750701

Sum of electronic and thermal Energies= -2137.712716

Sum of electronic and thermal Enthalpies= -2137.711772

Sum of electronic and thermal Free Energies= -2137.821735

E(RB3LYP) = -2138.40794093

Imaginary frequency = 190i

E(RB3LYP/6-311+G**) = -2138.91153758

0 1

C	-1.84435200	-1.64002000	1.84192400
C	-0.97765600	-0.80910600	2.60484800
C	0.40828500	-0.99040600	2.54053300
O	0.97669500	-1.91329500	1.86670600
C	-3.29452800	-1.81486100	2.28460800
C	-4.13701800	-1.85166900	1.03336300
C	-5.32780400	-1.62136600	-1.46040200
C	-3.41199400	-1.34318300	-0.05486700
C	-5.45830900	-2.25267500	0.87169000
C	-6.05671700	-2.13967900	-0.38753100
C	-3.99422200	-1.21074700	-1.32699800
H	0.32943800	5.73331500	-0.96676500
C	-0.39509700	5.00540200	-0.61552400
C	-2.22947400	3.07121700	0.25195500
C	-0.03646000	3.65740700	-0.58256900
C	-1.66680200	5.39040100	-0.20140500
C	-2.57352200	4.41868900	0.24049800
C	-0.94976700	2.67674100	-0.16821500
H	-1.95194100	6.43778900	-0.21918900
H	-3.56691300	4.71334800	0.56720400

H	-2.94305200	2.31654500	0.57118700
S	1.61890300	3.13060700	-0.96723200
N	-0.60527100	1.32676600	-0.20203600
H	-1.29228300	0.63251000	0.12402100
N	1.39621800	1.57656700	-1.48137900
C	0.46397900	0.84467500	-0.90781200
N	0.51857600	-0.51651200	-1.00846900
H	-0.31391800	-1.02330900	-0.70180100
O	2.13669800	3.93126400	-2.07841000
O	2.39322700	3.09435200	0.28869200
C	1.49625500	-1.19240600	-1.84790700
C	3.35125000	-2.90669100	-2.01690400
C	1.83296400	-2.51108200	-4.00953800
C	2.67767800	-3.53652500	-3.24715600
C	0.81951400	-1.83613500	-3.07710100
C	2.31113600	-2.26391500	-1.08278400
H	4.07008700	-2.14407300	-2.34658600
H	1.31117800	-2.99107000	-4.84573200
H	2.03875000	-4.36943700	-2.92043600
H	0.07836300	-2.57411400	-2.73612300
H	1.61848400	-3.03618000	-0.72503400
H	3.92016200	-3.67710600	-1.48680100
H	2.49030300	-1.74654100	-4.44694000
H	3.44559200	-3.96871900	-3.89915800
H	0.26780300	-1.05174000	-3.60599500
O	-2.13296300	-1.00130500	0.23526500
N	2.92031900	-1.72774600	0.20343400
C	3.71999800	-0.47005400	0.08619000
H	4.61867400	-0.65766800	-0.50707600
H	3.98824400	-0.16392700	1.09776500
H	3.12629000	0.32201400	-0.36850700
C	3.69570100	-2.76788200	0.94117300
H	4.65593500	-2.94960400	0.45585400
H	3.10730800	-3.68555700	0.98556300
H	3.84787600	-2.39537000	1.95278900
N	1.22313500	-0.14984900	3.36089200
C	1.06448900	1.30386500	3.32966200

O	2.61348100	-0.43126000	3.21562100
C	3.05322300	-1.13470000	4.37371000
C	-3.21020400	-0.65079600	-2.48661900
H	-2.30600400	-1.24045300	-2.68574200
H	-2.87849600	0.37835100	-2.29720200
H	-3.81294200	-0.64303500	-3.40035900
H	1.53372400	1.73304900	4.22011000
H	0.00176700	1.54565500	3.34447300
H	1.52142000	1.74774900	2.43528700
H	2.83572400	-0.56846700	5.28702700
H	4.13781600	-1.24270000	4.26288100
H	2.58422900	-2.12381600	4.43789000
H	-1.40600800	-0.05553900	3.25673200
H	-3.59063300	-0.97828500	2.93432000
H	-3.38888100	-2.72947600	2.88307400
H	-6.02020500	-2.64767500	1.71532400
H	-5.80208900	-1.53380000	-2.43605600
H	-7.08666400	-2.45335400	-0.53317700
H	-1.37804700	-2.56234700	1.50011000
H	2.07927900	-1.57088000	0.88804200
H	2.17594300	-0.41540400	-2.20628300

TS (catalyst A)

Zero-point vibrational energy 1716626.6 (Joules/Mol)

410.28361 (Kcal/Mol)

Zero-point correction= 0.653829 (Hartree/Particle)

Thermal correction to Energy= 0.690259

Thermal correction to Enthalpy= 0.691204

Thermal correction to Gibbs Free Energy= 0.583293

Sum of electronic and zero-point Energies= -1933.217289

Sum of electronic and thermal Energies= -1933.180858

Sum of electronic and thermal Enthalpies= -1933.179914

Sum of electronic and thermal Free Energies= -1933.287824

E(RB3LYP) = -1933.87111706

Imaginary frequency = 208i

E(RB3LYP/6-311+G**) = -1934.31737508

0 1

C	-1.22856100	-0.51622500	2.34154700
C	-0.72109200	0.80056000	2.39411800
C	0.65841400	1.02685200	2.27688200
O	1.53045500	0.10236400	2.21542800
C	-2.59735600	-0.83499400	2.92330400
C	-3.27114400	-1.82709700	2.00683700
C	-4.28989500	-3.41825400	-0.02187600
C	-2.65328900	-1.84125800	0.74432400
C	-4.38728500	-2.61763900	2.25684400
C	-4.89861100	-3.42428100	1.23540100
C	-3.16298600	-2.63427500	-0.30167300
H	-2.10635300	5.02369700	-3.19359200
C	-2.36989600	4.17112100	-2.57265100
C	-3.01666600	1.98445000	-0.99203300
C	-1.41514900	3.17334800	-2.36682200
C	-3.63941800	4.09178200	-2.00198500
C	-3.95547600	2.98752200	-1.20712500
C	-1.73475800	2.06309300	-1.57088700
H	-4.37211200	4.87540100	-2.17365300
H	-4.93858500	2.90167500	-0.75173300
H	-3.26881900	1.12743300	-0.37305700
N	-0.86818700	0.99383500	-1.25152900
H	-1.26285200	0.36535200	-0.53790500
C	0.37105400	0.63570600	-1.68144100
N	0.87786900	-0.42198900	-0.95369100
H	0.22165700	-0.86625200	-0.30505800
C	2.00519900	-1.23220000	-1.38688700
C	4.18525900	-2.35317900	-0.74562100
C	2.71649900	-3.40276200	-2.52798200
C	3.71743200	-3.66476200	-1.39855300
C	1.53877300	-2.55737000	-2.02848000
C	2.99037800	-1.53210800	-0.23285900
H	4.74830700	-1.76352800	-1.48199300
H	2.35073500	-4.35034600	-2.94077200
H	3.25011000	-4.30095900	-0.63333900

H	0.95368600	-3.12979700	-1.29421200
H	2.46219300	-2.10497900	0.54000000
H	4.87297200	-2.58720500	0.07362300
H	3.22063400	-2.87598000	-3.35038700
H	4.59006200	-4.21402800	-1.77101900
H	0.86198200	-2.30793000	-2.85241800
O	-1.57111400	-1.04453400	0.63190300
N	3.41642600	-0.27133800	0.50826200
C	3.92970200	0.84565300	-0.33881700
H	4.88357700	0.55991900	-0.78946500
H	4.05878700	1.71038500	0.31281800
H	3.20453000	1.09397000	-1.11566400
C	4.35950300	-0.55504100	1.62961800
H	5.35653200	-0.78026000	1.24803800
H	3.97314800	-1.38994000	2.21570100
H	4.38796600	0.33312500	2.25985300
N	1.11277800	2.38088400	2.33229000
C	0.50661700	3.39943100	1.47899300
O	2.52709400	2.48396400	2.18202900
C	3.10203800	2.71183300	3.46544500
C	-2.51404400	-2.62798600	-1.66219600
H	-1.47517700	-2.97900900	-1.61634100
H	-2.48498600	-1.62017700	-2.09545500
H	-3.05582700	-3.27623700	-2.35856200
H	0.78530400	4.38934100	1.85156500
H	-0.57780600	3.30169300	1.51423600
H	0.83442600	3.30335700	0.43470700
H	2.67822600	3.60697700	3.93594400
H	4.17230900	2.86479200	3.28847800
H	2.95243300	1.84840300	4.12417500
H	-1.40839700	1.63053600	2.51504100
H	-3.18985700	0.08799200	3.00815500
H	-2.47588800	-1.22588400	3.94200600
H	-4.86019600	-2.60459800	3.23679500
H	-4.69733500	-4.04145400	-0.81592900
H	-5.76761600	-4.05106600	1.41565400
H	-0.48332600	-1.29197800	2.50032000

H	2.53880400	0.06344000	1.05638100
H	2.51839700	-0.65205900	-2.15875800
H	-0.43808100	3.24733900	-2.82108000
S	1.24820600	1.36645100	-2.93861400
